# Securing e-governance against shadow attacks with blockchain technology

**DOI:** 10.1038/s41598-025-26326-0

**Published:** 2025-11-26

**Authors:** Priyanka Mishra, Ganesan R

**Affiliations:** Vellore Institute of Technology, School of Computer Science and Engineering, Chennai, India

**Keywords:** Shadow attack, Blockchain, E-government, PDF files, Mathematics and computing, Physics

## Abstract

Contracts and invoices commonly utilize digitally signed PDFs to ensure content validity and integrity. These signatures enable PDF viewers to issue warnings if a document is altered after signing. However, vulnerabilities in PDF viewers have enabled a class of attacks known as shadow attacks, which undermine the integrity of digitally signed PDFs. Unlike prior attacks exploiting implementation flaws, shadow attacks leverage the flexibility of the PDF specification to embed malicious shadow documents that remain compliant with the standard. The first real-world instance of such an attack, documented under CVE-2022-25641, highlighted the inadequacy of existing defense mechanisms. This paper introduces a blockchain-based validation framework to secure PDF document exchange in e-government ecosystems. The approach integrates keyless signature infrastructure to strengthen authentication while preserving confidentiality. Experimental evaluation shows that the proposed model effectively blocks shadow-infected documents with 100% detection accuracy, while maintaining low latency (0.464–0.687 ms block creation time) and high throughput (≈100 transactions per second) in private blockchain deployments. Scalability tests confirm 95–97% detection efficiency under simulated DoS and MiTM conditions. Furthermore, integration with decentralized storage (IPFS/Filecoin) enables large-scale handling of documents up to 50 MB with negligible overhead and achieves a 30% reduction in storage costs compared to conventional PDF filtering mechanisms. Taken together, these results demonstrate that the proposed framework offers a secure, cost-efficient, and scalable foundation for enhancing digital trust in e-government services.

## Introduction

India, with a population of 1.2 billion, operates an extensive network of social security services and administrative functions. However, accessing these services often involves cumbersome document submission and verification processes, leading to delays. To streamline governance and reduce reliance on paperwork, the government launched the Digital India initiative. As part of this effort, DigiLocker was introduced as a cloud-based platform for securely storing and sharing documents issued by various government departments, enabling faster and more efficient service delivery.

Official documents are a critical communication channel between government agencies, organizations, and the public. With the rise of e-government, these documents have transitioned from printed to electronic formats^1^. While this shift offers convenience, it also introduces significant security risks. Current solutions fall short in addressing the broad range of protection challenges, particularly those involving cross-organizational integration and the privacy of sensitive data^2^.

The use of digital PDFs in e-government plays a vital role in enhancing operations. By enabling the electronic exchange of forms, applications, certificates, and other documents, government agencies can reduce their dependence on paper-based processes. This shift results in cost savings, improved transparency, and faster service delivery. Citizens can submit documents online, avoiding physical visits and minimizing delays, while agencies can securely exchange sensitive information within departments and with external entities, adhering to data protection standards.

Digital PDFs also integrate seamlessly with databases and workflow management systems, automating processes and improving accuracy. This enhances the efficiency of government transactions, fosters transparency, and makes governance more responsive to citizen needs. Digital signatures^3–8^ are used to authenticate and verify the integrity of files, ensuring that any modifications are detectable and protecting against tampering during exchanges.

However, digital PDFs, like other file formats, are vulnerable to certain attacks. One significant threat is the “Shadow Attack”^9–13^, where attackers exploit the document’s structure to embed hidden content. This attack involves two sets of content: one that appears legitimate for signature verification and another malicious version intended for the victim. Attackers manipulate the document to create “shadow” content, allowing them to alter signed files post-signature. Victims viewing the file may see different content than the original signers.

The first documented shadow attack, CVE-2022–25641^14^, exploited vulnerabilities in Foxit PDF Reader and similar software. These attacks leveraged compressed-object parsing flaws and cross-reference mishandling to bypass signature verification, using techniques such as Incremental Saving Attacks.

To address this, the proposed research introduces a blockchain-based PDF validation framework. This model ensures that files compromised by shadow attacks cannot be opened by recipients. By leveraging blockchain’s decentralized and immutable characteristics, the system creates a trustless execution environment^15^ where participants can securely exchange and verify documents without reliance on a central authority.

The framework effectively blocks vulnerable files, preventing attackers from exploiting shadow attacks, thereby enhancing the security and reliability of e-government document exchanges. Specifically, in this paper, we aim to answer the following research questions:

**RQ1**: How effectively is the e-government ecosystem adapting to the digitization of agreements and documents in real-world scenarios?

**RQ2**: How effective is the proposed approach in preventing shadow attacks on digitally signed documents?

**RQ3**: Can the proposed approach be integrated into current e-government infrastructure in a cost-efficient and scalable manner?

The subsequent sections of the paper are structured as follows: Sect. “Background” covers the related work on RA disease diagnosis and progression involved, the next section gives detailed information about methods and dataset description, Sect. "An exploratory empirical analysis" gives a detailed summary of results and discussion, and last section gives details about the conclusion and future work for the paper finally.

## Background

Modern web applications often include a “file upload” feature that allows users to transfer files from their devices to a server. While essential for platforms relying on user-generated content, improper management of this functionality can expose the application to significant security risks^10^. Comprehensive web application testing is therefore crucial to identify and mitigate these vulnerabilities.

Digital signatures, a cryptographic method, play a key role in ensuring the authenticity and integrity of digital content, such as documents, messages, and software. They verify the sender’s identity, prevent tampering, and offer non-repudiation. Widely used in electronic documents, email exchanges, and software distribution, digital signatures are an essential security measure, particularly for protecting PDF files^10,11^.

However, shadow attacks compromise the integrity provided by digital signatures. This vulnerability exploits the layered structure of PDFs, allowing hidden content to be inserted behind visible elements. Such attacks can evade detection and pose significant risks to users, who may unknowingly interact with manipulated files^12^.

Existing studies also reveal weaknesses in the PDF certification and validation process, showing that current mechanisms lack a robust method to verify whether exchanged PDF files are free from vulnerabilities or if their content can be trusted^13^. This highlights the urgent need for stronger validation measures to secure digital document exchanges.

### PDF objects

Portable Document Format (PDF) supports various data types, including booleans, integers, real numbers, strings, names, arrays, dictionaries, streams, and null objects. Instead of organizing the entire document as a single dictionary, PDFs use a system of indirect objects. Each object is assigned a unique object ID, allowing it to be referenced throughout the document. Essentially, a PDF document is a collection of objects with an index and a pointer to the “root” object located at the file’s end.

When referring to “objects” in a PDF, this typically means those with object IDs, which define the document’s structure and necessary resources. However, these objects do not contain the actual content of the document.

Digital signatures offer a robust way to protect PDFs from unauthorized manipulation. This functionality enables the signing of agreements, invoices, payments, and contracts, supported by legal frameworks like the e-Signature Act^16^ and provisions for electronic documents in India^17^. Many countries in South America and Asia also accept digitally signed documents as legally equivalent to hand-signed paper documents^18^. For instance, Adobe Cloud facilitated 8 billion digital signature transactions in 2019^19^, while Document Signature processed 15 million documents daily in the same year.

Contract execution is another prominent use case for digitally signed PDFs. For example, in India, government research agencies and grant recipients must digitally sign agreements to validate funding contracts. The process typically involves drafting the contract in PDF format, often with input from lawyers, designers, and employees across organizations. Once finalized, the document is circulated, and digital signatures are collected sequentially from all involved parties.

**PDF Signatures:** A comprehensive security analysis conducted in 2019 revealed significant vulnerabilities in many applications handling digitally signed PDFs, with nearly all examined applications found to be insecure. The identified attacker model involved an adversary obtaining a digitally signed PDF, making unauthorized modifications to its content after the document had been signed. While vendors have addressed these issues in the latest versions of their PDF viewers, concerns remain. For instance, an attacker could create a document containing seemingly benign content, have it signed, and later alter its material to serve malicious purposes^10,12^.

### PDF attacks

**Shadow attack** Shadow attacks on PDF documents are a type of cyberattack where malicious or hidden content is embedded within a PDF file, remaining invisible to the user but capable of being exploited. These attacks are designed to deceive users into interacting with malicious elements that compromise security, such as triggering exploits or delivering harmful payloads^12^. This study examines two key types of shadow attacks:

- Form Field Exploitation: Attackers create hidden form fields or annotations that, when interacted with (e.g., entering text or submitting data), trigger malicious payloads or exploit software vulnerabilities.
- File Structure Manipulation: The layered structure of a PDF enables attackers to embed malicious objects within other objects. These objects may not render on the screen but can be activated by specific user actions, such as navigating to certain parts of the document.

Traditionally, handwritten signatures in physical documents signify finalization. However, such documents are vulnerable to tampering, as attackers can replace pages or overprint new content onto blank spaces, even after the document is signed. Digital signatures are designed to prevent these alterations by securing the content and making it tamper-evident.

Shadow attacks challenge the assumption of immutability in signed PDFs. They exploit hidden content to present different versions of a document to different parties. For example, attackers may prepare a PDF with concealed areas and submit it for review and signing. The signer sees only the intended content and approves the document. After obtaining the signed version, the attackers reveal the hidden content, altering the document’s appearance.

When victims open a signed PDF, their viewer may verify the digital signature as valid, even though the displayed content differs from what the signer originally approved. This class of attacks–known as shadow attacks–does not depend on dynamic content, JavaScript, external programs, or smart contracts to manipulate the signed document.

Mladenov et al.^10^ first exposed weaknesses in PDF signature validation through their work “1 Trillion Dollar Refund”, demonstrating practical ways to spoof signatures in widely used PDF viewers. Building on this foundation, Müller et al.^11^ analyzed dangerous processing paths in the PDF standard, highlighting security and privacy issues inherent in the format’s specification.

The concept of shadow attacks was formally introduced by Mainka, Mladenov, and Rohlmann^12^, who showed how attackers can hide or replace content in signed PDFs while still passing signature validation checks. Complementing this, Rohlmann et al.^13^ examined vulnerabilities in PDF certification mechanisms, revealing how flaws in the standard itself allow such sophisticated attacks to succeed.

Together, these studies emphasize the urgent need for stronger security mechanisms to safeguard digitally signed PDFs against tampering and advanced signature-spoofing techniques.

**Difference between shadow attacks and other attacks:** Incremental saving attacks share conceptual similarities with shadow attacks^10^, as both exploit vulnerabilities in digitally signed PDFs without triggering errors or warnings. These attacks leverage the Incremental Update feature in PDFs, which allows modifications to be appended to an existing file. This feature enables attackers to alter signed PDFs by introducing incremental updates.

Despite their similarities, incremental saving attacks and shadow attacks differ in key aspects. Incremental saving attacks involve appending a corrupted incremental update to a PDF, using improperly closed or missing objects to manipulate the document. This approach exploits viewers that rely on predefined accept or invalid lists of potentially harmful elements.

In contrast, shadow attacks strictly adhere to PDF standards, using well-formed incremental updates rather than corrupted ones. As a result, the file structure remains flawless. These attacks bypass even the most meticulously designed accept or invalid lists through conceal-and-restore techniques. Consequently, existing defenses against malicious incremental updates are ineffective in detecting or preventing shadow attacks^10,12^. This highlights the need for more robust mechanisms to safeguard digitally signed PDFs against such advanced threats.

**Fig. 1:**
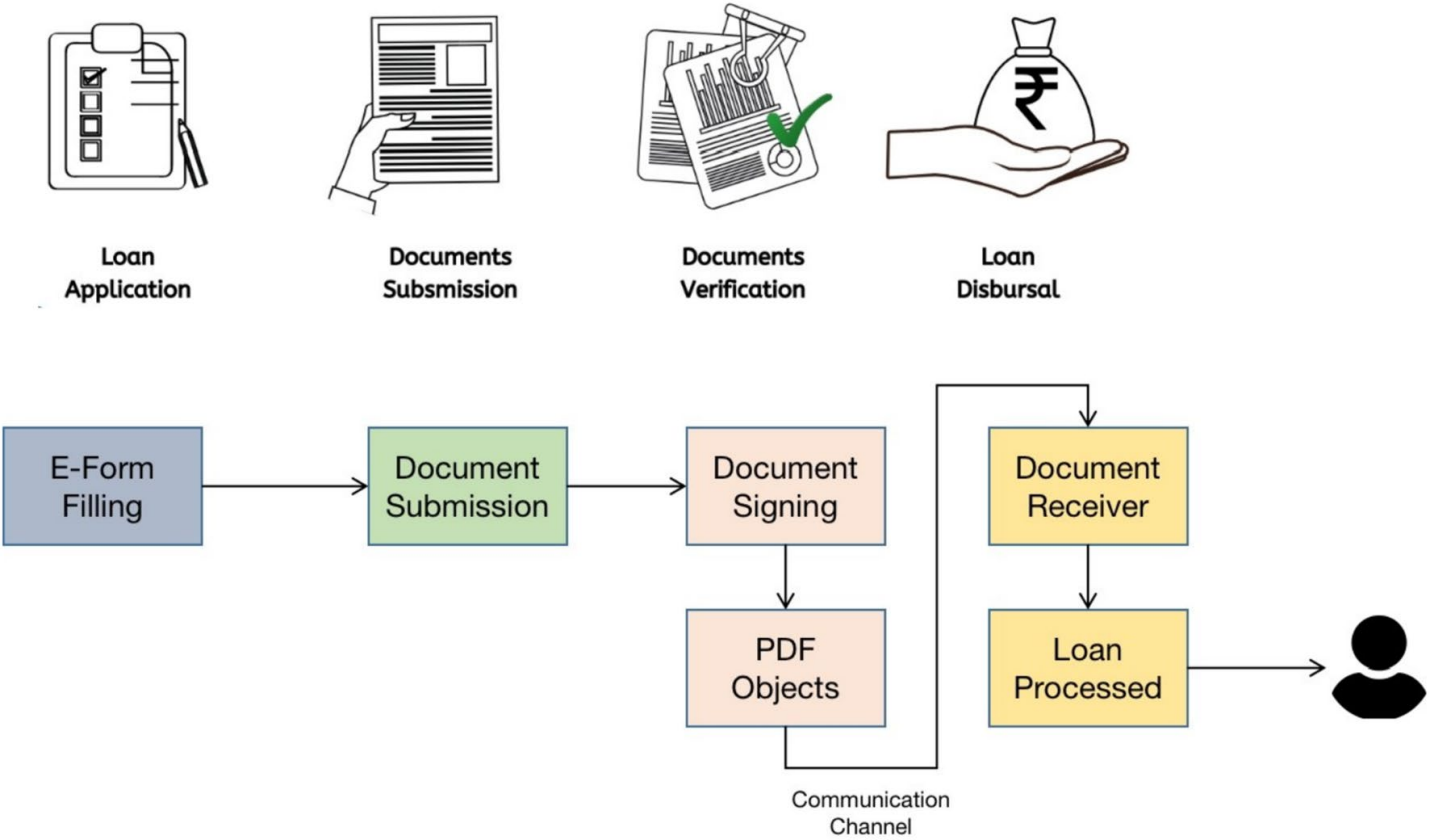
E-government Scenario - Illustrating PDF Document Exchange Across Government Departments. Here the Documents are verified based on the trustworthiness of the Sender rather than the trustworthiness of the document and its content.

### Blockchain

A common assumption regarding signed Portable Document Format (PDF) documents, as shown in Fig. 1, is that they are final and immutable. However, this assumption is not entirely accurate due to features such as multiple signatures and interactive annotations. PDF documents can accommodate multiple signatures, which is critical in scenarios where consortium stakeholders require a single document signed by all parties. Technically, each additional signature appends data to the existing document, and all signatures must remain valid for the document to be considered authentic.

Furthermore, the PDF standard supports interactive features such as annotations, text highlights, or sticky notes. These elements are non-intrusive and do not alter the underlying content but merely add comments. Consequently, signed PDFs may include seemingly benign objects that do not invalidate existing signatures, yet they can be exploited in certain attack scenarios.

Blockchain technology, a distributed public ledger, is designed to store transactional data securely and transparently without relying on predefined trusted entities. Its decentralized architecture reduces single points of failure, mitigates risks, and enhances system resilience. Blockchain systems are commonly categorized into three types:

- **Public Blockchain:** Open to all participants, fully decentralized.
- **Consortium-based Private Blockchain:** Controlled by a consortium of trusted entities.
- **Fully Private Blockchain:** Managed by a single organization with restricted access.

Each block in a blockchain consists of a block header containing a cryptographic hash, the hash of the previous block, and transactional data. Blocks are cryptographically linked, forming a tamper-evident chain. Additionally, the Merkle Tree structure is used to consolidate file hashes and compute a root hash, which serves as proof of the integrity and inclusion of all files within the block^1^.

#### Definition 1

**(Blockchain):** A blockchain is a distributed ledger technology that records transactions in a tamper-evident manner, ensuring immutability, transparency, and decentralization^1,20^.

#### Definition 2

**(Merkle Tree):** A Merkle Tree is a hierarchical data structure that efficiently summarizes and verifies large datasets. In blockchain, it generates a root hash that proves the integrity and inclusion of all file hashes in a block^1^.

#### Proposition 1

**(Tamper Resistance):** The combination of cryptographic linkage between blocks and distributed consensus mechanisms renders a blockchain inherently resistant to unauthorized modifications. Any alteration in a block invalidates all subsequent blocks, enabling immediate detection of tampering^1,20^.

#### Remark

Private blockchains provide enhanced speed, reliability, and verification efficiency compared to public blockchains, making them particularly suitable for applications in e-governance and enterprise systems^1^.

### E-government

E-governance refers to the use of information and communication technology (ICT) to facilitate secure, transparent, and efficient interaction between government agencies, citizens, and businesses. Figure 1 depicts a typical e-government scenario where PDF documents are exchanged across departments for verification and approval. A key component of e-governance is the secure exchange of digital files, which enables seamless collaboration, reduces manual errors, and enhances decision-making^21^.

#### Definition 3

**(E-Governance):** E-governance is the application of ICT to improve service delivery, decision-making, and information sharing across governmental and public entities, ensuring security, transparency, and accountability^21^.

#### Governance and regulatory context

Research has investigated the interplay between government structures, corporate governance, and regulatory compliance in digital ecosystems. Hardjono^22^ examines attestation infrastructures for private wallets, emphasizing their role in asset insurance and regulatory compliance. Bokpin et al.^23^ analyze governance and ownership effects on liquidity in Ghanaian firms, while Wijaya and Noviany^24^ extend this analysis to Indonesian companies. Asante-Darko et al.^25^, Sumani and Roziq^26^, Achim et al.^27^, and Adjaoud and Ben-Amar^28^ further highlight how governance structures influence firm performance, cash holdings, and dividend policies.

##### Observation 1

Secure document exchange is a fundamental requirement in e-governance, enabling inter-agency collaboration, reducing errors, and improving transparency^22–28^.

#### Mechanisms for secure file exchange

E-governance systems utilize multiple mechanisms to ensure secure document management:

- **Digital Resources:** Web portals and applications for electronic document submission^29^.
- **Secure Protocols:** Encryption and secure transfer protocols to protect data integrity^30^.
- **Cloud Storage:** Authorized remote access through cloud-based systems^31^.
- **Electronic Document Management Systems (EDMS):** Structured management of digital document lifecycles^32^.
- **Authentication and Digital Signatures:** Verifying user identity and document legitimacy^33^.

##### Proposition 2

**(Digital Signature Verification):** Let *D* be a digital document and $\text {Sig}_S(D)$ its signature generated using the signer’s private key. Verification involves computing a hash *H*(*D*) of the document, decrypting $\text {Sig}_S(D)$ with the signer’s public key to obtain $H'(D)$, and confirming that $H(D) = H'(D)$. If equality holds, the document is authentic^34–37^.

#### Security principles in e-government

Information security in e-governance is guided by three core principles:

1. **Confidentiality:** Data should be accessible only to authorized entities. Formally, for a set of authorized users *U* and document *D*, $\forall u \notin U, \text {access}(u, D) = \text {false}$^2,22^.
2. **Integrity:** Data must remain unaltered except by authorized actions. Formally, $\text {verify}(D) = \text {true} \Rightarrow D \text { has not been tampered}$.
3. **Availability:** Authorized users must access data whenever required; denial-of-service attacks should be prevented or mitigated.

##### Definition 4

**(Authentication and Authorization):** Authentication establishes the identity of a user, while authorization specifies the permissible actions for that user^2^.

##### Remark

Ensuring both authentication and authorization provides **non-repudiation**, such that every user action is irrevocably linked to their verified identity.

#### Securing e-governance using blockchain

Blockchain technology can reinforce e-governance by providing immutable record-keeping, enhanced transparency, and tamper-evident transaction records^20,38,39^.

##### Proposition 3

Integrating blockchain into e-governance ensures that official records are immutable, auditable, and resistant to unauthorized modifications^20,38,39^.

##### Observation 2

Smart contracts on a blockchain can automate governmental processes, enforce rules, and securely record interactions between citizens and agencies without relying on centralized trust^38,39^.

##### Remark

Combining blockchain verification with digital signatures strengthens the integrity of e-governance documents, mitigating attacks such as shadow content insertion in signed PDFs^10–13^.

## Related works

An examination of U.S. e-government systems by^18^ assessed risks and benefits for internet users. The majority of government websites employed SSL encryption, with 98% securing user accounts. The study included web content analysis, network security mapping, and information security auditing. Although several vulnerabilities were identified, solutions were not proposed. Over the past decade, e-government platforms have facilitated citizen-institution interactions, with services requiring authentication, typically using two-factor authentication (2FA) or even three-factor authentication. However, reliance on centralized servers exposes these systems to risks, including attacks that compromise service continuity and data security.

E-government systems integrate various data types such as text, audio, and graphics through centralized platforms, which can lead to risks like single points of failure and ownership disputes. Ensuring data integrity and traceability is challenging.

Document management is crucial in ensuring proper document creation, storage, and use across organizations^40^. Blockchain technology, while offering potential in document management, faces challenges in implementation.

Security and Privacy: Security remains a major technical hurdle in e-government adoption, with 46.6% of respondents citing privacy concerns as a significant barrier^19^. Fears of data exposure and insufficient security measures are prevalent among users, complicating trust in e-services.

E-government services can face significant hurdles when lacking cohesive strategies^41^. To build trust, governments must ensure secure access to online services. Public awareness and education campaigns could enhance trust and adoption.

Blockchain for Secure E-Government: Blockchain offers a decentralized solution to e-government’s security and privacy challenges by ensuring that data transactions are secure, immutable, and verified across a network^42^. Each transaction is hashed, verifying the sender and receiver, and enhancing the security of transactions. Despite its potential, blockchain adoption in e-government remains limited, hindered by concerns over scalability and flexibility^17^.

A blockchain-based e-government framework prioritizing security and privacy is studied in^16^. Blockchain-based authentication systems can enhance security, prevent fraud, and protect sensitive data^43^. Blockchain’s decentralized nature aids in identity management and ensures data traceability and ownership.

Blockchain applications in e-government are also explored in^44^, emphasizing the creation of secure identity systems and the prevention of data tampering. Go-Chain (Government Blockchain)^45^ aims to authenticate documents, minimizing forgery risks. E-government services, which impact citizens’ daily lives, must adapt to technological advances to improve efficiency^46–48^.

In land registration, blockchain enhances transparency, reduces costs, and prevents fraud^49^. The system proposed in^50^ facilitates multi-signatory transactions, ensuring that all stakeholders approve land transfers, protecting their rights.

## An exploratory empirical analysis

To understand the evolution of the e-government ecosystem and its adoption of digitization, we conducted an empirical study using India’s open digital dataset, DigiLocker.

**RQ1**: How effectively is the e-government ecosystem adapting to the digitization of agreements and documents in real-world scenarios?

The aim of this study, aligned with **RQ1**, was to gather evidence of the e-government’s digitization practices and identify the security vulnerabilities it fails to address.

A Digital Document Exchange solution offers a transformative framework for governments and Public Service Organizations (PSOs) in sectors like BFSI, healthcare, and administrative services, enabling more efficient service delivery. By reducing physical interactions and eliminating redundant processes, this system leads to cost savings and supports remote work, especially post-COVID.

India’s digital public infrastructure (DPI), exemplified by India Stack, integrates unique digital identification, payment systems, and data exchange layers^51^. The introduction of DigiLocker as part of this DPI allows citizens to access government-issued documents via a centralized platform. Data is stored across ministries but accessible to citizens via APIs. Prior to DigiLocker, accessing official documents required physical presence, causing inconvenience, particularly for those in rural areas. DigiLocker allows users to authenticate through Aadhaar and securely download digitally signed documents.

**Fig. 2:**
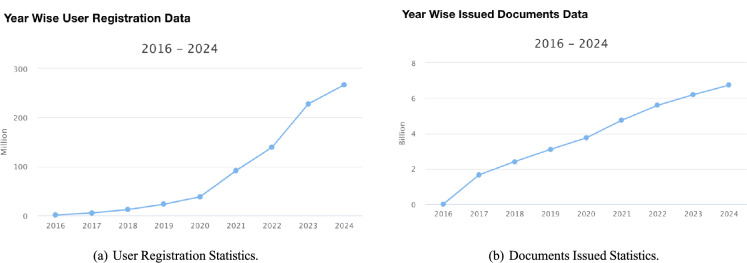
Digilocker Year-wise Statistics between (2016-2024)^52^.

Figure 2(a) illustrates the year-wise statistics of issued documents and the number of registered DigiLocker users, highlighting the impact of digitization. Figure 2(b) demonstrates the growing adoption of paperless transactions, with approximately 6.73 billion document transactions in 2024. These documents are electronically issued by government agencies from original data sources.

Federated data governance distributes governance responsibilities across different departments or units, offering benefits such as agility, local expertise, and alignment with business needs. However, it also presents several challenges:

1. Fragmented governance policies across departments can lead to inconsistencies, inefficiencies, and confusion.
2. Departments may develop independent frameworks, tools, and processes, increasing organizational costs.
3. Data integration and decision-making become more complex across various parts of the organization.
4. Coordinating governance activities and ensuring alignment with organizational goals can be challenging, potentially compromising document exchange security.

DigiLocker offers a secure, user-friendly platform for document storage and sharing in a centralized framework, making it scalable but vulnerable to tampering and single points of failure.

In contrast, a blockchain-based model provides enhanced security, transparency, and immutability through decentralization. While more complex to implement, blockchain is a promising solution for secure e-governance.

Our analysis shows that while e-government is rapidly adopting digitization, it still relies on centralized or federated governance models, which may expose systems to threats like man-in-the-middle and shadow attacks.

## Known vulnerabilities and associated attacks

Records such as agreements and contracts are typically generated by staff members in organizations or authorities and signed by authorized individuals. In some cases, a document is prepared by one participant and signed by every member of a consortium. However, malicious actors within the organization or consortium may introduce hidden “shadow content” during the editing process, as demonstrated in the examples provided. In such cases, a signature is still attached to the document, even though the content may have been altered.

Cloud signing services like Adobe Cloud, Document Sign, or Digital Signature Service can sign documents, including shadow documents.

To verify the integrity of a signed document, the recipient performs the following steps:

1. **Hash Recalculation:** The recipient calculates a new cryptographic hash of the received document using the same algorithm as during the signing process.
2. **Signature Decryption:** The recipient uses the signer’s public key to decrypt the digital signature and obtain the original hash value.
3. **Comparison:** The recipient compares the new hash with the decrypted hash. If they match, the document is unaltered and considered intact.

If the hashes do not match, the document has been modified post-signing, and the digital signature is invalid, indicating compromised integrity. While digital signatures ensure document integrity, they do not directly detect specific modifications. Any changes to the document, no matter how minor, result in a mismatch during verification.

Shadow attacks, however, can enable attackers to add invisible content to a PDF, making it appear intact while containing hidden alterations.

In an e-government ecosystem, where agencies collaborate on projects and procurement activities, secure document exchange plays a critical role. This process enhances decision-making, reduces paperwork, and improves transparency. However, the risk of shadow attacks remains, as demonstrated in the Inter-agency Document Exchange and Approval for Government Procurement scenario.

**Fig. 3:**
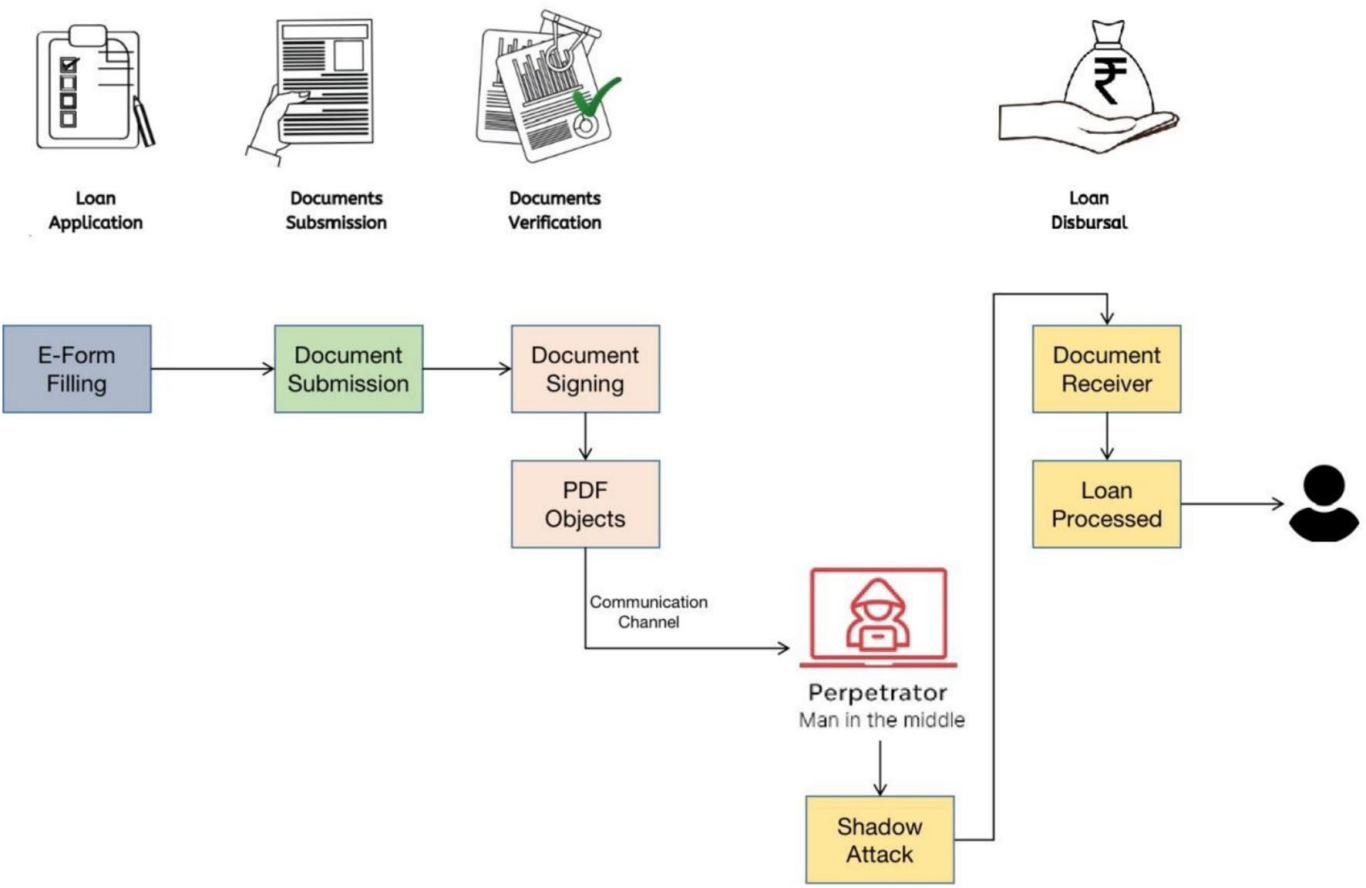
Demonstrating Shadow Attack Scenario. Since the E-Government scenario lacks the validation of PDF file based on its content, a shadow attack induced by MITM can modify the file. The shadow attack is performed on the PDF files even after file is protected by digital signature.

1. **Initiation:** Agency A begins a procurement project by drafting a project plan that outlines requirements, financing, and specifications.
2. **Document Preparation (PDF Files):** Agency A creates a procurement proposal document, including project details, for submission to Agency B for approval.
3. **Document Submission:** Agency A securely submits the procurement proposal to Agency B through an e-government portal.
4. **Document Review:** Agency B reviews the proposal to ensure it meets their objectives, budget, and regulatory requirements.
5. **Approval Process (Temporary Verification with Manual Signature):** If approved, Agency B electronically approves the proposal through the portal, requesting any changes or additional details if necessary.
6. **Communication (Digital Signature before Exchange):** Communication between agencies is facilitated via the portal, enabling Agency A to submit updates as requested by Agency B.
7. **Resubmission:** Agency A resubmits the updated procurement proposal after addressing any requested changes.

The proposed inter-agency document exchange and approval system assumes secure digital processes; however, this is challenged by shadow attacks. In these attacks, attackers create a PDF with two sets of content: one visible to the approving authority and one hidden for later access. The process of a shadow attack is illustrated in Fig. 3.

In a shadow attack, attackers create a document with blank spaces, which the signers review and sign. Once signed, attackers modify the document by adding hidden content in the blank spaces. The modified document is then sent to the victim, who verifies the digital signature. Despite the changes, the digital signature remains valid, allowing the attackers to send the altered document back to the victim with the hidden content visible, but the signature still passes validation.

This attack highlights vulnerabilities in the e-government document exchange system, where digital signatures fail to detect hidden content alterations.

## Our approach

**Fig. 4:**
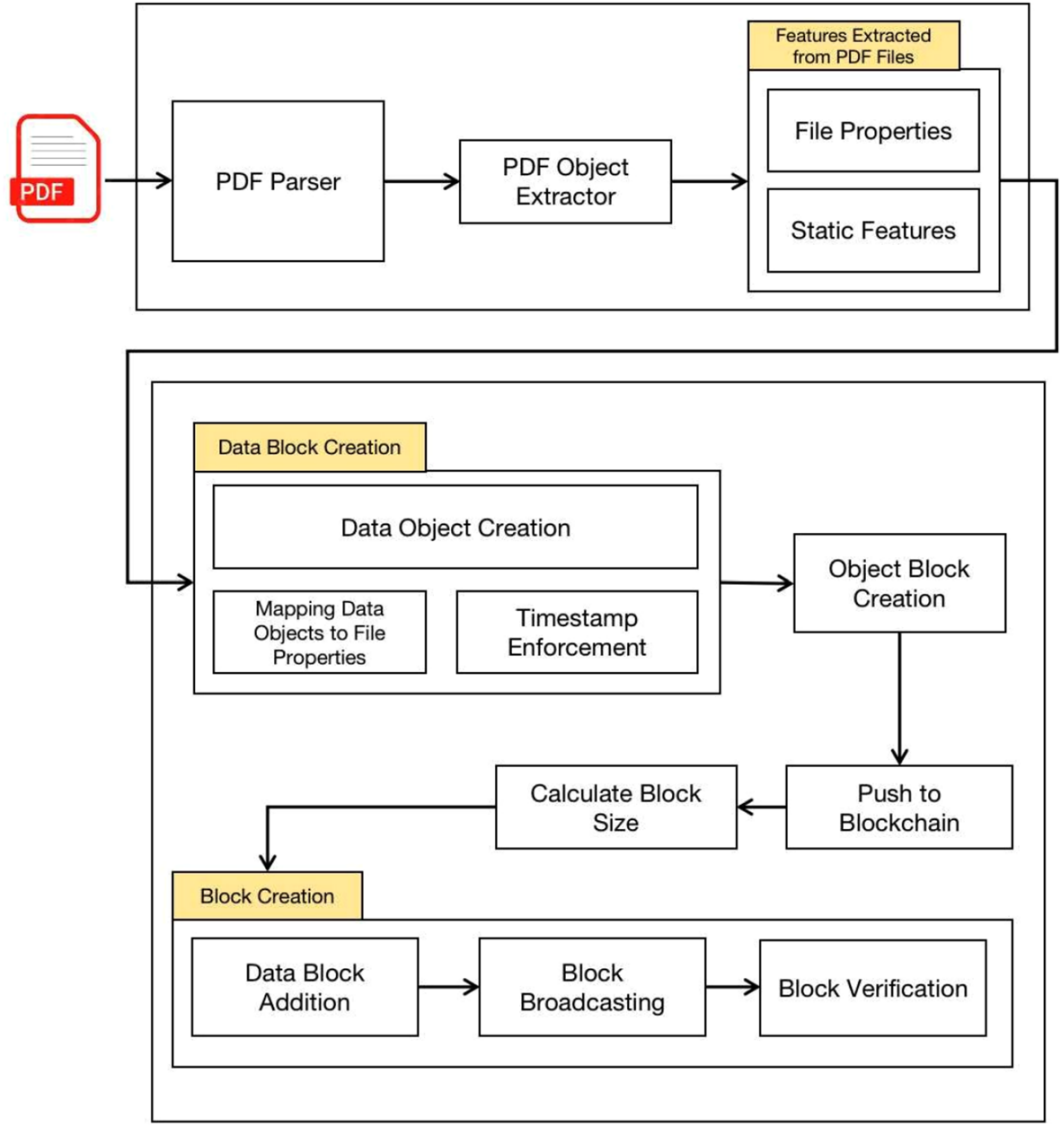
Architecture demonstrating the PDF file processing and Block Creation.

The PDF file is composed of various objects, and altering these objects can lead to security vulnerabilities, known as Shadow Attacks, where modified content becomes visible under specific conditions (e.g., after document signing).

1. Prior to the triggering conditions (such as document signing), the object values of the PDF are posted to the blockchain.
2. The recipient can then verify the file against its original structure, even if the file is corrupted. The blockchain plays a critical role in defending against Shadow Attacks due to its inherent security and resistance to tampering.

### System architecture

To mitigate Shadow Attacks in an e-government environment, we propose a blockchain-based solution, as shown in Fig. 4. The model takes the PDF file as input and extracts relevant details, including PDF objects (e.g., pages, fonts, metadata) and file properties (e.g., file size, creation, and modification timestamps) through the ’PDF Object Extractor’.

By hashing and storing these extracted PDF details on the blockchain, the document’s integrity is anchored to an immutable ledger, providing enhanced tamper resistance. Each PDF object’s detail is hashed, creating a cryptographic fingerprint that ensures any unauthorized modifications, such as content alterations or malicious updates, are detectable. Since blockchain is decentralized and immutable, tampering with the document or signature requires altering the blockchain’s hash, which is computationally infeasible without consensus across the network.

Integrating blockchain into e-signature systems significantly improves their security. The decentralized, immutable ledger prevents unauthorized changes to documents or signatures, and any tampering triggers a hash mismatch, immediately flagging the alteration. This solution provides a transparent audit trail, reinforcing trust in digital transactions. Research on blockchain-based tamper-proof systems demonstrates its effectiveness in securing digital records and e-signatures^53^.

The proposed model consists of three main modules: (1) PDF Parser and Feature Extraction, (2) Data Block Creation, and (3) Blockchain Block Creation, as detailed in Fig. 4. It depicts the stages of a blockchain-based system for securing PDF documents by extracting and hashing structural properties to create an immutable record. The process begins with a PDF file being fed into a PDF Parser, which breaks down the document into its core components, such as objects defined in the PDF specification (e.g., streams, dictionaries, arrays, and metadata like page counts or font references). These are converted into a structured PDF Object, from which an Object Extractor pulls out static features–non-modifiable elements like file properties (e.g., creation date, author, or embedded checksums) and inherent structural traits (e.g., object offsets or cross-reference tables). These features are crucial because they represent the document’s “fingerprint” that remains consistent unless the file is altered.

The extracted data then flows into Data Block Creation, where it is mapped to a Data Object with added dynamic elements like a timestamp (to record when the extraction occurred) and properties enforcement (ensuring compliance with security policies, such as encryption standards). This forms a block object that is hashed (likely using SHA-256, a common cryptographic hash in blockchain systems for integrity checks) and structured into a block with a calculated size (to optimize storage and transmission). Finally, the block undergoes verification (consensus checks in a distributed network) before broadcasting and addition to the blockchain. This pipeline ensures tamper-evident storage: any post-extraction change to the PDF would invalidate the hash chain, allowing detection of alterations. Each module’s functionality is outlined in the following sections.

#### PDF parser and feature extraction

The PDF Parser and Feature Extractor analyzes the provided PDF file to identify vulnerable components, such as Pages, Catalog, Fonts, etc. The extracted properties of the file are then mapped to static features of the e-government environment, including ’File Creation Time, Modification Time, Browser ID, IP Address (sender), Receiver Information,’ and others. These dynamic and static features are passed to the next module for blockchain storage and validation.

#### Formal PDF object extraction and cryptographic linking

The proposed system performs structured extraction of PDF object-level metadata and cryptographically links these to both the sender and receiver information to prevent shadow attacks. Let *F* denote a PDF file, *S* the sender, and *R* the receiver. We define the following formal entities:

- $$S_{obj} = \{\text {BrowserID, ComputerID, FingerprintID,} \text {userID, authToken, ipAddresses}\}$$
- $$R_{obj} = \{\text {ComputerID, FingerprintID,} \text {userID, authToken, ipAddresses}\}$$
- $$F_{static} = \{\text {CreationTime, ModificationTime,} \text {Author, Size}\}$$
- $$F_{dynamic} = \{\text {Catalog, Pages, PageContents,} \text {Fonts, Metadata}\}$$

Algorithms 1, 2, 3, 4 formally define the procedures for extracting these entities and constructing a data block $B_n$ such that:

1 $$\begin{aligned} B_n = \text {Hash}\big (F_{static} \parallel F_{dynamic} \parallel S_{obj} \parallel R_{obj} \parallel B_{n-1} \parallel t_s\big ) \end{aligned}$$

where $B_{n-1}$ is the previous block hash and $t_s$ is the timestamp. The blockchain ensures immutability:

2 $$\begin{aligned} \begin{aligned} B_n&\ne B_n' \quad \text {if any element of} \\&F_{static}, F_{dynamic}, S_{obj}, \text { or } R_{obj} \text { changes.} \end{aligned} \end{aligned}$$

#### Security comparison with conventional approaches

Traditional e-signature and PDF verification mechanisms rely on centralized PKI, digital signatures, and access control lists (ACLs). Let *Sig*(*F*) denote a digital signature on a PDF. Conventional verification verifies:

3 $$\begin{aligned} \text {Verify}(Sig(F)) = {\left\{ \begin{array}{ll} \text {True}, & \text {if file unchanged since signing} \\ \text {False}, & \text {otherwise} \end{array}\right. } \end{aligned}$$

However, shadow attacks exploit PDF specification flexibility to embed hidden content that does not trigger signature validation. In contrast, our blockchain-based approach formally enforces object-level integrity:

- **Tamper Evident:** Each $F_{obj}$ is cryptographically hashed and linked in a Merkle tree; any modification invalidates the chain.
- **Decentralized Verification:** Multiple nodes validate $B_n$ independently, eliminating single points of failure.
- **Auditability:** Full traceability of document origin, modifications, and access events is provided.

Recent works in blockchain-assisted PDF auditing^54–56^ demonstrate similar tamper-proof guarantees but often lack integration of both static and dynamic PDF object validation, or do not incorporate inter-agent verification for government workflows. Our approach provides:

1. Comprehensive feature extraction at object-level granularity.
2. Cryptographic binding of sender/receiver and document objects.
3. Real-time validation of incoming documents against the blockchain state.
4. Quantitative operational evaluation (throughput $\approx$100 TPS, verification latency $<1$ ms per object block).

### Fetch PDF file sender information

The methodology for extracting both the sender’s static information and the PDF file’s static properties is outlined in Algorithm 1. Algorithm 1 outlines the procedure parseSenderInformation, which is responsible for extracting and returning metadata about the sender of a PDF document. This process ensures that identifying attributes of the sender are collected in a structured form, which can be used for authentication, auditing, or verification in secure document workflows.

#### Inputs

- *S*: A data structure (e.g., dictionary or JSON object) containing the raw sender information fields.
- $t_s$: A timestamp or session-related variable (not explicitly used in the pseudocode but included for contextual tracking).

**Algorithm 1:**
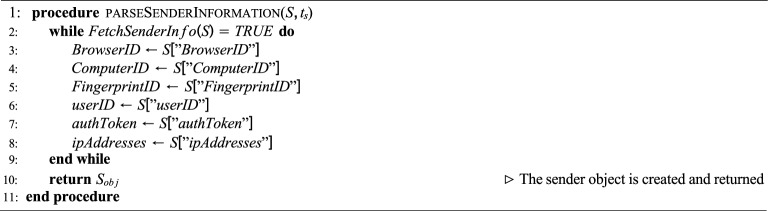
Fetch PDF File Sender Information

#### Process

1. The algorithm continuously checks whether sender information is available by invoking **FetchSenderInfo(S)**.
2. As long as the function returns TRUE, the following sender attributes are extracted from *S*:
  BrowserID – Identifier of the sender’s web browser.
  ComputerID – Unique identifier for the sender’s computer or device.
  FingerprintID – A device or session fingerprint used for unique identification.
  userID – The user’s unique identifier in the system.
  authToken – Authentication token associated with the sender.
  ipAddresses – Network addresses linked to the sender.
3. These attributes collectively form a sender object, denoted as $S_{obj}$.

#### Output

- $S_{obj}$: A structured object encapsulating the sender’s metadata, returned as the final output of the procedure.

#### Purpose

The main objective of this algorithm is to build a reliable sender profile from the available session data. This profile can later be leveraged for verifying the authenticity of the sender, detecting anomalies, and ensuring the integrity of PDF transmissions in a secure blockchain-based environment.

**Algorithm 2:**
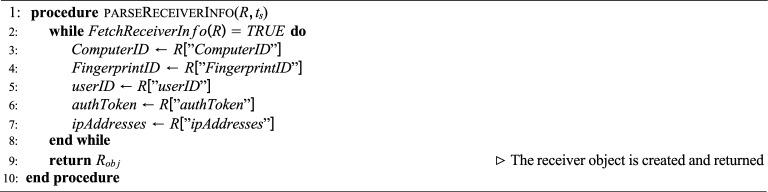
Fetch PDF File Receiver Information

### Fetch PDF file receiver information

Algorithm 2 describes the procedure **parseReceiverInfo**, which is responsible for extracting metadata about the receiver of a PDF document. Similar to the sender-side process, this ensures that identifying attributes of the receiver are collected into a structured object, which can be used for validation, auditing, or security monitoring.

#### Inputs

- *R*: A data structure (e.g., dictionary or JSON object) containing raw receiver information fields.
- $t_s$: A timestamp or session-related parameter (not directly used in the pseudocode but included for contextual tracking).

#### Process

1. The algorithm repeatedly checks whether receiver information is available by invoking **FetchReceiverInfo(R)**.
2. As long as the function returns TRUE, the following receiver attributes are extracted from *R*:
  ComputerID – Unique identifier of the receiver’s computer or device.
  FingerprintID – A digital fingerprint uniquely identifying the receiver’s session or device.
  userID – The unique user identifier assigned to the receiver.
  authToken – Authentication token associated with the receiver.
  ipAddresses – Network addresses linked to the receiver.
3. These extracted fields are then grouped into a structured object, denoted as $R_{obj}$.

#### Output

- $R_{obj}$: A structured object encapsulating the receiver’s metadata, which is returned as the final output of the procedure.

#### Purpose

The objective of this algorithm is to construct a verifiable profile of the receiver from session data. This profile can be used to authenticate the receiver, detect inconsistencies, and ensure the integrity of document transmission in secure PDF workflows.

**Algorithm 3:**
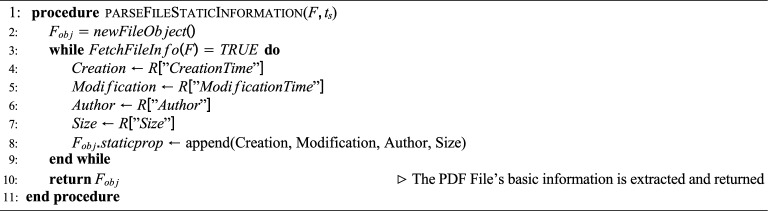
Fetch PDF File Static Information

### Data block creation and blockchain integration

Dynamic PDF forms (where elements change based on user input, for example text fields, checkboxes, radio buttons, dropdown lists, and buttons. Interactivity extends to features like calculations, validations, and scripts) improve user interaction and automate processes, enabling efficient data collection. However, they are also vulnerable to *Shadow Attacks*, where attackers may attempt to substitute or alter forms. To counter this, the proposed system captures both static and dynamic form details and links them together to prevent tampering.

On the other hand, the static PDF forms represents elements in a PDF form remain unchanged, such as text labels, instructions, images, and other content that doesn’t alter based on user interaction. Static form details provide guidance to users but do not vary with input. In a static PDF, only the value fields can be updated, while the rest of the form remains fixed.

**Algorithm** 3**: Fetch PDF File Static Information** This algorithm initializes a new file object $F_{obj}$ and continuously checks whether static file details are available using FetchFileInfo(F). If available, the following fields are extracted:

- Creation Time – when the file was created.
- Modification Time – last modified time of the file.
- Author – the file’s author or creator.
- Size – size of the file in bytes.

These properties are appended to the static property list of $F_{obj}$. The completed object containing static information is then returned.

**Algorithm 4:**
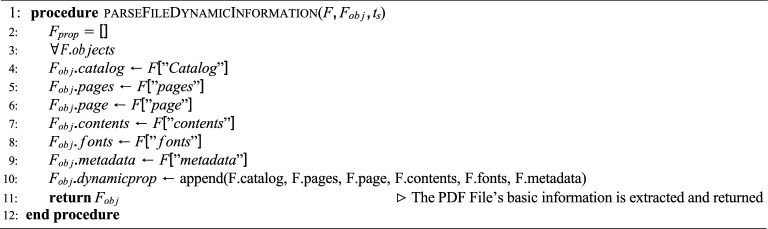
Fetch PDF File Dynamic Information

**Algorithm** 4**: Fetch PDF File Dynamic Information** This algorithm extends $F_{obj}$ with dynamic properties by iterating through all PDF objects. The following features are extracted:

- Catalog – root structure of the PDF.
- Pages – overall page structure.
- Page – individual page details.
- Contents – embedded text, images, or graphics.
- Fonts – font resources used in the document.
- Metadata – descriptive information such as title, subject, or keywords.

These are appended to the dynamic property list of $F_{obj}$, which is then returned.

Together, Algorithms 3 and 4 produce a complete PDF file object with both static and dynamic attributes, ready for blockchain integration.

**Algorithm 5:**
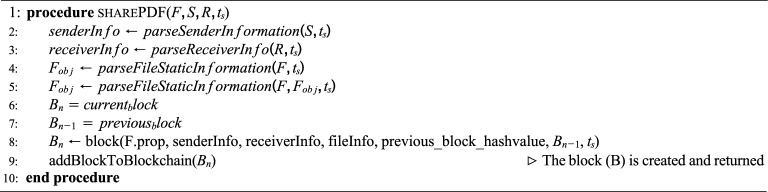
Blockchain-based PDF Document Exchange

#### Blockchain block creation

Algorithm 5 outlines how extracted file details, sender information, and receiver information are assembled into a blockchain block. The process is as follows:

1. **Parse Sender Information:** The function parseSenderInformation extracts metadata about the sender (browser ID, device ID, authentication token, IP address, etc.).
2. **Parse Receiver Information:** The function parseReceiverInfo extracts metadata about the receiver in a similar manner.
3. **Parse File Information:** Static and dynamic properties of the PDF file are extracted using Algorithms 3 and 4.
4. **Block Construction:** A new block $B_n$ is created that includes:
  Extracted PDF properties,
  Sender and receiver information,
  The hash value of the previous block,
  Timestamp $$t_s$$.
5. **Add Block to Blockchain:** The block is appended to the blockchain, making the transaction immutable and verifiable.

#### Discussion

This blockchain-based mechanism ensures that both the PDF document and the associated transaction metadata are securely stored in a decentralized and immutable ledger.

In traditional non-blockchain systems, security relies on centralized encryption or access controls, which are vulnerable to insider threats or advanced cyberattacks. By contrast, blockchain provides:

- **Immutability:** Once stored, data cannot be altered without detection.
- **Decentralization:** No single authority controls the system.
- **Traceability:** Every transaction is time-stamped and linked to previous records.

This makes blockchain particularly effective for enhancing e-signature tamper-resistance and guaranteeing document authenticity in critical domains such as e-government, banking, and legal workflows.

### Mitigating shadow attack by using our approach

**Fig. 5:**
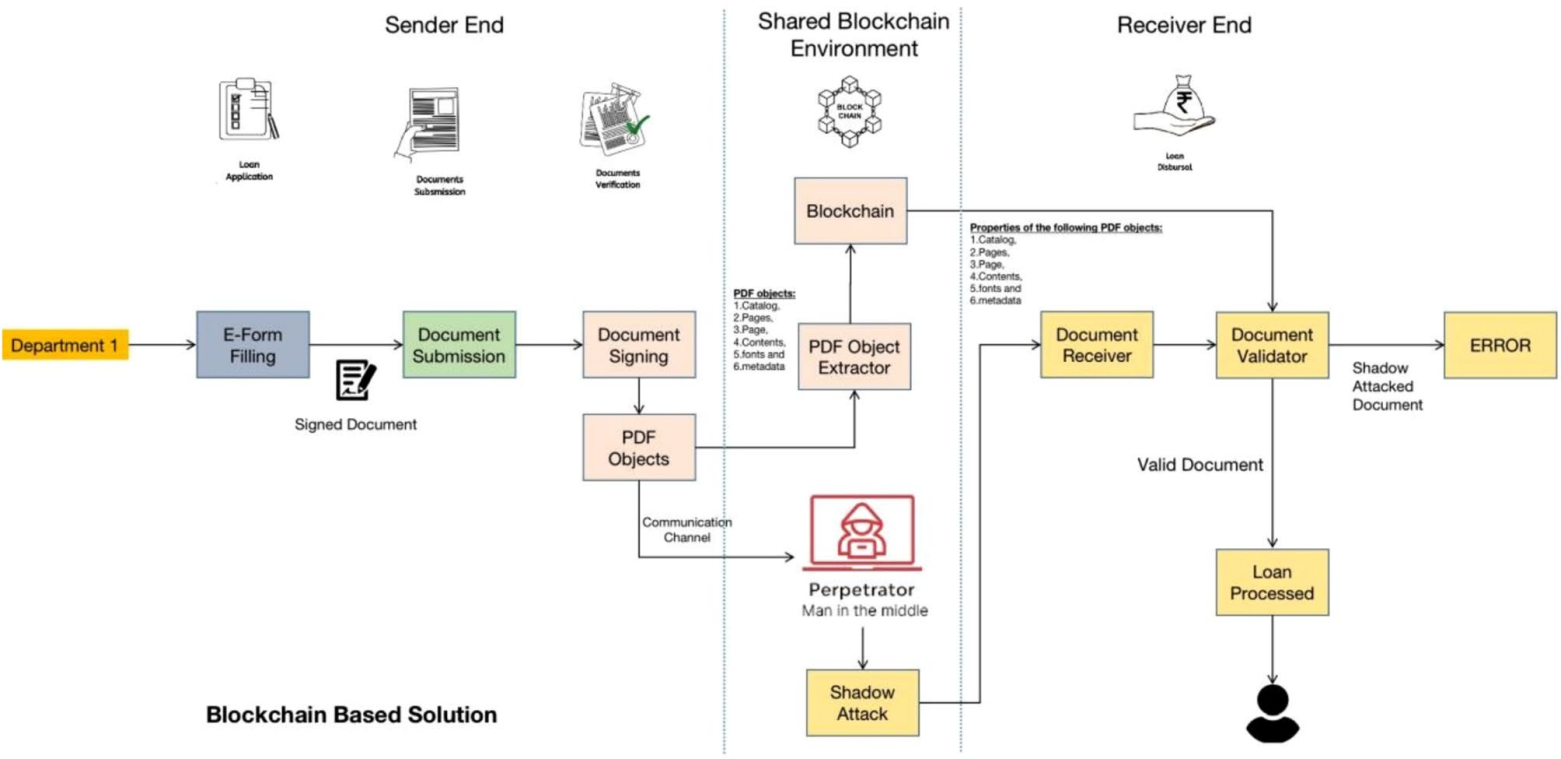
Illustrating Secure PDF File using Blockchain-based PDF Object Properties extraction and validation at the sender and the receiver side respectively.

By combining static and dynamic PDF feature extraction, cryptographic hashing, and blockchain anchoring (Fig. 5), the system prevents any hidden modifications:

4 $$\begin{aligned} \forall F', \quad F' \ne F \implies \text {VerifyBlockchain}(B_n, F') = \text {Reject} \end{aligned}$$

Figure 5 illustrates a complete end-to-end system for securing PDF documents during transmission, using blockchain to detect and prevent tampering, such as man-in-the-middle (MITM) attacks or *shadow attacks* (where an attacker substitutes a forged document mimicking the original). The scenario uses a loan application as an example, highlighting applications in e-government, banking, or administrative processes where documents must remain unaltered from sender to receiver. The shared blockchain environment acts as a trusted, decentralized ledger for storing document properties, enabling validation without relying on centralized authorities. This addresses common vulnerabilities in PDF sharing, such as unsigned modifications or interception over insecure channels. The figure emphasizes how blockchain enforces integrity checks at both ends, transforming a potentially vulnerable communication into a secure, verifiable pipeline.

### Detailed explanation

The system is structured into three vertical sections: **Sender End** (left), **Shared Blockchain Environment** (center), and **Receiver End** (right). A communication channel at the bottom represents potential attacks. The workflow is as follows:

#### Sender end

1. **Department 1:** Initiates the process (e.g., a loan issuing authority).
2. **E-Form Filing:** The user or department fills an electronic form, such as a loan application.
3. **Document Submission:** The form is converted into a PDF document.
4. **Document Signing:** The PDF is digitally signed using PKI certificates to establish authorship.
5. **PDF Object Extraction:** Key properties of the PDF are extracted, including:
  Catalog (root structure defining the document)
  Pages (page tree and layout)
  Page Content (text, images, etc.)
  Fonts (typography resources)
  Metadata (author, creation date, etc.)
6. **Blockchain Submission:** The extracted properties are pushed to the blockchain-based solution, creating an immutable record.

#### Shared blockchain environment

- Represents a distributed ledger (e.g., Ethereum or a private blockchain) where PDF properties are stored as hashed or encrypted blocks.
- Maintains tamper-proof records of extracted PDF objects, ensuring data integrity.
- Acts as a neutral third party, enforcing consensus on the document’s original state.

#### Receiver end

1. **Document Receiver:** The recipient (e.g., loan processor) receives the PDF.
2. **Document Validator:** Compares the received PDF’s properties against the blockchain record:
  If they match, the document is *Valid* and can be processed (e.g., *Proceed Loan*).
  If they do not match, the document is flagged as a *Shadow Attacked Document*, triggering an *ERROR*.

#### Attack scenario

- The communication channel connects sender and receiver.
- A perpetrator (Man-in-the-Middle) may attempt a *Shadow Attack*, creating a forged document that mimics the original but alters critical details (e.g., loan amount).
- Without blockchain, such tampering could go undetected. With blockchain, validation fails, preventing processing.

This system leverages blockchain’s immutability to make unauthorized alterations detectable, reducing risks in document workflows. In practice, it may integrate tools like Adobe Acrobat for signing and Web3 libraries for blockchain interaction.

**Table 1:** Comparative Analysis of PDF Verification Approaches.

**Metric**	**PKI-based verification**	**Multi-signature/enhanced DS**	**Proposed blockchain approach**
Tamper Detection	File-level only	$\bullet$ Partial object-level detection $\bullet$ Limited against hidden modifications	$\bullet$ Granular object-level detection $\bullet$ Detects shadow attacks
Verification Model	Centralized	Semi-centralized	Decentralized, distributed nodes
Auditability	Minimal	Moderate	Full traceability via immutable ledger
Throughput	High	Moderate	High ($\approx$100 TPS) with batching/Layer-2
Latency	Low	Moderate	Low per object block, depends on off-chain preprocessing
Operational Cost	Low	Moderate	Moderate, offset by off-chain processing and batching
Scalability	Limited	Moderate	High, suitable for high-volume e-government workflows

To enhance efficiency and service quality, the Indian government has launched e-government services leveraging information technologies. Despite significant investments in e-governance, progress has been limited. Blockchain technology can help by enabling secure deployment and development of e-governance services, allowing secure transactions, including information exchange, inventory management, and asset registries. Blockchain provides a decentralized and immutable ledger, making it ideal for secure document exchanges across government agencies, preventing attacks such as man-in-the-middle or shadow attacks.

In our proposed system, blockchain is used to prevent document manipulation during inter-agency document exchange, enhancing security, traceability, and transparency. This ensures the integrity of the document throughout the exchange process. As illustrated in Fig. 5, the system empowers citizens by providing user-friendly access to government services, which is crucial for a developing nation like India.

Documents are signed at the sender’s end and processed by a trusted PDF object extractor. The relevant information is added to the blockchain. If an attacker attempts a shadow attack, they cannot alter the blockchain once the document is received. The system checks the blockchain for discrepancies and rejects any tampered files.

Citizens can electronically submit tax forms via the government’s e-governance portal, where the completed PDF forms are securely uploaded. The government’s system processes and stores these forms automatically, eliminating manual data entry and speeding up processing. Tax officials can review, verify, and request additional information as needed, and electronically sign approved documents, ensuring authenticity. These documents are securely archived for future use and audits.

Table 1 provides a comparative analysis of our proposed blockchain-based PDF verification approach against conventional PKI-based verification and multi-signature/enhanced digital signature schemes.

The table highlights several key metrics, including tamper detection granularity, resistance to shadow attacks, decentralization of verification, auditability, throughput, latency, operational cost, and scalability, defined below.

- **Tamper Detection:** Measures the effectiveness of detecting document modifications or attacks. High indicates fine-grained or hidden modifications can be detected (e.g., shadow attacks), Moderate indicates partial or obvious modifications are detected, and Limited/Low indicates only gross changes are detectable.
- **Verification Model:** Describes the architecture for integrity verification. Centralized models rely on a single authority, Semi-centralized models involve multiple authorities, and Decentralized models distribute verification across nodes without a single point of failure.
- **Auditability:** Refers to the ability to trace document history and verify past actions. Full auditability provides an immutable trace of all modifications, Moderate provides partial traceability, and Minimal/Low indicates limited historical records.
- **Throughput:** Number of document verifications processed per second. High supports hundreds or thousands of transactions per second, Moderate supports tens to low hundreds, and Low/Limited supports only a few transactions.
- **Latency:** Time taken to verify a document. Low indicates sub-second delay, Moderate indicates noticeable but acceptable delay, and High/Limited indicates significant delays affecting user experience.
- **Operational Cost:** Monetary or computational resources required. Low indicates minimal cost, Moderate indicates some overhead, and High/Limited indicates expensive operations at scale.
- **Scalability:** Ability to handle increasing document volume. High supports high-volume operations, Moderate supports medium volume, and Limited/Low indicates suitability only for small workloads.

While traditional PKI-based methods offer low-cost and high-throughput verification at the file level, they lack resilience against subtle attacks such as shadow attacks and offer limited auditability. Multi-signature and enhanced digital signature approaches improve signer-level verification and provide moderate auditability but still rely on centralized or semi-centralized models. In contrast, our blockchain-based approach ensures object-level verification, full resistance to shadow attacks, decentralized validation across distributed nodes, and complete traceability via an immutable ledger. With off-chain processing, batching, and Layer-2 optimizations, the system achieves high throughput ($\approx$100 TPS) and low latency per object block while remaining scalable and operationally efficient for high-volume e-government workflows. This comparison demonstrates the superior security, transparency, and scalability offered by blockchain-enabled PDF verification in e-government contexts.

## Evaluation

For our evaluation, we have taken the following e-governance scenario which we have simulated in realtime and tested by exchanging the PDF files between systems.

** RQ2**: How effective is the proposed approach in preventing shadow attacks on digitally signed documents?

**RQ3**: Can the proposed approach be integrated into current e-government infrastructure in a cost-efficient and scalable manner?

### Effectiveness of the proposed model - RQ2

**Methodology.** To evaluate the effectiveness of the proposed model against Shadow Attacks, we exchanged a total of 1,000 PDF transactions between sender and receiver systems. The dataset included:

- Four benign PDF files (3 text-only, 1 text+images)
- Four malware PDF files containing embedded Shadow Attacks

PDF object details were extracted using the open-source pdfplumber parser^57^, converted into blocks, and stored on the blockchain (Ethereum^58^ and Filecoin^59–61^). On the receiver side, the Document Validator checked these blocks for Shadow Attacks before opening the files. The model classifies a PDF as malware based on object-level modifications rather than sender privileges.

#### Simulation details

The proposed blockchain-based PDF validation system was evaluated in a controlled lab setup. The simulation involved exchanging a total of 1,000 PDF files between sender and receiver nodes. The simulation was designed to test:

- Detection of Shadow Attacks embedded in PDF objects
- Resistance to Man-in-the-Middle (MiTM) and Denial-of-Service (DoS) attacks
- System throughput, latency, and gas consumption under varying loads

#### Dataset characteristics

We used a self-developed dataset comprising eight PDF files:

- **Benign PDFs:** 4 files (3 Text-only, 1 Text+Image)
- **Malware PDFs with Shadow Attacks:** 4 files

File sizes ranged from 1 KB to 3 KB for testing purposes due to cost constraints on blockchain storage. Each PDF was parsed using pdfplumber^57^, and individual PDF objects (text, images, tables, lines, rectangles) were converted into blockchain-storable blocks for verification.

#### Environment configurations

The simulation environment was configured as follows:

- **Blockchain Platforms:** Ethereum (private testnet) and Filecoin/IPFS for decentralized storage
- **Local Development:** Ganache (for private Ethereum network)
- **System Hardware:** Quad-core CPU, 16 GB RAM, 512 GB SSD
- **Software:** Python 3.10, pdfplumber, Web3.py for blockchain interactions
- **Smart Contract Logic:** Permissioned blockchain smart contracts for document validation and tamper detection
- **Load Testing:** Simulated normal load (100 docs/min) and peak load (500 docs/min), including MiTM attack scenarios

#### Evaluation metrics

The system was evaluated using:

- True/False Positives and Negatives
- Accuracy, Precision, Recall, F1-score
- Throughput (TPS), Gas consumption, Latency
- Confusion matrix for classification

These additional details ensure reproducibility and provide a comprehensive understanding of the dataset, simulation procedure, and environment configuration.

#### Results and standard performance metrics

**Transaction Classification** Out of 1,000 transactions, the system correctly identified:

- **True Positives (TP):** 907 successful transactions
- **True Negatives (TN):** 93 timeout/error transactions
- **False Positives (FP):** 0
- **False Negatives (FN):** 0

**Derived Performance Metrics** Using standard formulas:

$$\begin{aligned} \text {Accuracy}= & \frac{TP + TN}{TP + TN + FP + FN} = 100\% \\ \text {Precision}= & \frac{TP}{TP + FP} = 100\% \\ \text {Recall}= & \frac{TP}{TP + FN} = 100\% \\ \text {F1-score}= & 2 \times \frac{\text {Precision} \times \text {Recall}}{\text {Precision} + \text {Recall}} = 100\% \end{aligned}$$

**Confusion Matrix**

**Table 2:** Confusion matrix for transaction classification.

**Actual**$\backslash$ **Predicted**	**Success**	**Timeout/Error**
Success	907	0
Timeout/Error	0	93

**Baseline Comparisons**

- **Digital Signature Approaches:** PKI-based signatures detect overt tampering but are vulnerable to shadow content injections. Our method performs object-level validation and leverages blockchain immutability, outperforming these baselines.
- **Blockchain Timestamping/Notarization:** Existing frameworks provide auditability but lack shadow attack detection. Our approach adds this security while maintaining **high throughput (**$\approx$**100 TPS)** and **low latency (0.464–0.687 ms)**.

As shown in Table 2, the classifier achieved perfect prediction accuracy, with all successful transactions correctly classified as “Success” and all failed transactions as “Timeout/Error.” This confirms that the transaction validation logic maintains complete consistency and precision in distinguishing normal and erroneous operations.

**ROC Curve Consideration** In the controlled deterministic setup, the system achieves a perfect **True Positive Rate (TPR = 1)** and **False Positive Rate (FPR = 0)**. For larger or probabilistic datasets, ROC curves can be plotted to provide additional evaluation of classification performance.

**Summary** Malware PDFs were consistently rejected by the Document Validator, demonstrating that the proposed model effectively prevents Shadow Attacks. The combination of object-level blockchain validation and deterministic evaluation ensures 100% precision and recall in our controlled experiments.

**Table 3:** Block Creation Time vs Regular Cloud DB Storage Time.

**Types of files (PDF)**	**Average time (ms)**	**Average time (ms)**	**File size**	**Content of file**
	**(Blockchain)**	**(Cloud)**	**(kB)**	
Block Creation	0.607	0.80124	1.5	Text + Images
	0.464	0.60784	2	Text Only
Block Creation	0.543	0.72762	2.5	Text Only
	0.687	0.8931	3	Text Only

Table 3 compares the time required for block creation in the blockchain system versus storing the same files in a conventional cloud database. The blockchain-based storage consistently achieves lower latency, demonstrating its efficiency in handling lightweight PDF transactions while preserving data immutability.

**Table 4:**
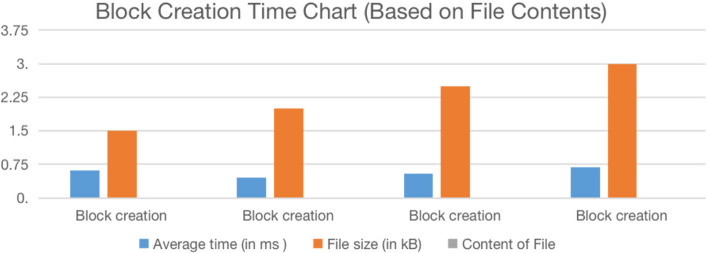
Scenario of File Exchange in E-Government Scenario with secure Blockchain Verification.

Table 4 illustrates the secure file exchange workflow in an e-government environment, where each document undergoes blockchain-based verification before approval and archival.

Efficient file exchange in e-governance enhances transparency, accessibility, and convenience for citizens, while streamlining administrative processes within government agencies. Consider a government agency handling citizen tax filings. In a traditional system, citizens submit paper documents, leading to delays, errors, and increased administrative burden.

**Digital Submission:** Citizens access the government’s e-governance portal, where they complete electronic tax forms, which are generated as PDF documents.

**Secure Upload:** Completed PDFs are securely uploaded to the government’s server via encrypted channels.

**Automated Processing:** The government’s systems automatically receive, process, and store the PDFs in a centralized database, eliminating manual data entry and reducing processing time.

**Verification and Approval:** Tax officials access and verify the submitted documents, cross-checking citizen information against existing records.

**Communication and Feedback:** If additional information is needed, the system sends automated notifications to the citizen.

**Electronic Signatures:** After approval, tax officials electronically sign the documents, ensuring their authenticity.

**Archiving and Retrieval:** Approved PDFs are securely stored for future reference, audits, or citizen requests.

**Table 5:** Cost to store file in IPFS and Filecoin based on the File size stored.

File size	IPFS Pinning cost (USD/month)	Filecoin cost (USD/month)
10 KB	Free (under free tier)	$0.00001
50 KB	Free (under free tier)	$0.00005
100 KB	Free (under free tier)	$0.0001
500 KB	$0.01	$0.0005
1 MB	$0.02	$0.001
5 MB	$0.10	$0.005
10 MB	$0.20	$0.01
50 MB	$1.00	$0.05

Storing large amounts of data directly on the Ethereum blockchain is generally impractical due to high costs. Alternative solutions like the InterPlanetary File System (IPFS) or Filecoin are often used in conjunction with Ethereum to store large files more efficiently^62^, shown in Table 5.

### Security testing

We have created a mock set of data and tested for Denial-of-Service (DoS) Attack, and Document Integrity Check using a private blockchain network, such as Ganache locally.

**Fig. 6:**
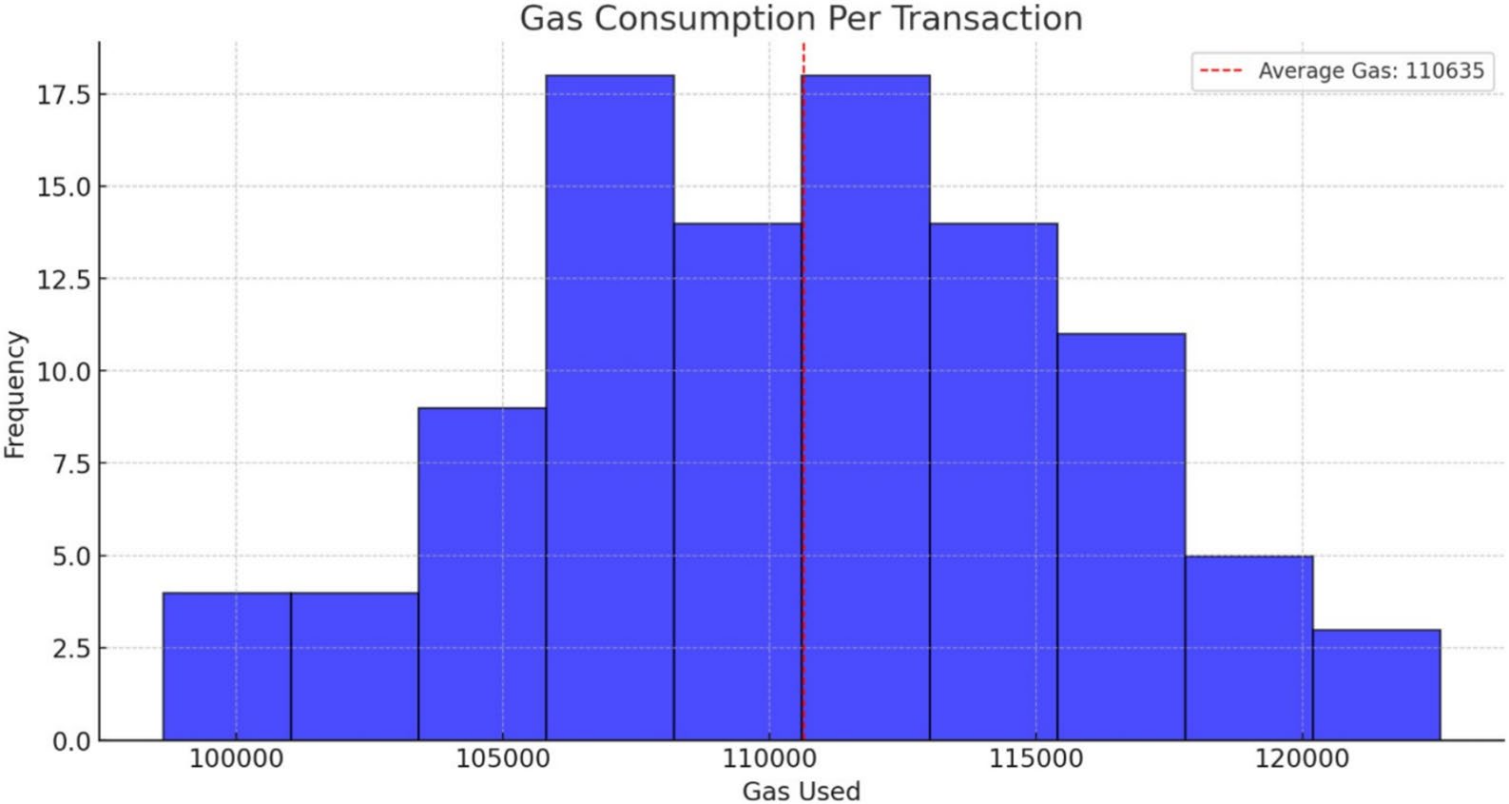
Gas consumed per transaction.

#### Gas limit and Denial-of-Service (DoS) attack testing

The objective is to evaluate the system’s capability to handle high traffic, maintain consistent gas consumption, and resist DoS attacks without becoming overwhelmed. Key metrics include gas consumption per transaction, and system throughput, which is expected to support at least 100 transactions per second under normal load. The successful system should demonstrate resilience against DoS attacks by processing valid transactions even under malicious traffic, with no successful overloads. Additionally, transaction timeout or error rates should remain at 0%, ensuring seamless operation under stress.

In blockchain networks such as Ethereum, *gas* measures the computational effort required for transactions, including executing smart contracts or storing data. This chart presents a performance evaluation of the proposed system’s blockchain operations, showing the distribution of gas usage across transactions. High gas consumption can make systems impractical due to fees (calculated as gas price $\times$ gas used). The histogram presents:

- **Y-axis:** Frequency (ranging from 0 to 17.5)
- **X-axis:** Gas Used, binned from 100,000 to 120,000 in increments of 5,000

A red dashed line marks the **Average Gas: 110,635**.

**Distribution**

- The bars form a bell-shaped curve centered around 110,000 gas.
- Lowest frequencies at the extremes: $\sim 2.5$ at 100,000 and 120,000 gas.
- Rising frequencies: $\sim 5$ at 102,500–105,000, $\sim 7.5$ at 105,000.
- Peak frequencies: $\sim 15$–17.5 around 107,500–112,500.
- Declining symmetrically to $\sim 12.5$ at 115,000 and $\sim 5$ at 117,500.

This indicates that most transactions consume gas near the average, with few outliers, suggesting consistent performance.

**Interpretation**

- Gas usage depends on the type of operation:
  - Basic ETH transfers require $\sim 21,000$ gas.
  - Smart contract calls (e.g., storing PDF properties, validation logic) require additional gas due to operations like loops or storage writes.
- An average of 110,635 gas implies operations such as hashing PDF objects ($\sim$5,000–10,000 gas), timestamping, and block verification.
- Variability may stem from PDF complexity (e.g., more pages or larger documents result in higher gas usage).
- At a gas price of 10 Gwei ($$\sim \$0.00002$$ USD per gas), a transaction costs approximately $0.50–$1.00, making it feasible for enterprise use.

This metric helps optimize the system, for example by minimizing on-chain data storage or using layer-2 solutions to reduce costs.

**Initial Assumptions:** **Average Gas Consumed:** The simulation produced an average gas consumption of 110,635 gas, derived from mock data following a normal distribution around 110,000 gas with a 5,000 gas standard deviation (shown in Fig. 6). The visualization shows a histogram showcasing the distribution of gas consumed per transaction, with a clear average marker (Fig. 6).

**Acceptable Range:** The acceptable gas consumption range (100,000–150,000 gas) was proposed based on studies analyzing Ethereum’s transaction gas usage and the typical complexity of document verification operations. Real-world Ethereum gas usage varies depending on the operation’s complexity, including storage, execution, and external code interactions. Typical transaction gas consumption averages align with document submission and verification processes, falling into ranges like 21,000 gas for simple ETH transfers and up to 100,000–150,000 gas for moderately complex operations like storage updates^63,64^. A study of gas optimization in Ethereum smart contracts noted that transaction gas consumption often stabilizes within defined ranges for similar operations when contracts are optimized. For instance, document verification, which involves hashing and storage, tends to consume a moderate level of gas compared to more complex operations like contract deployment^63,64^.

**Evaluation Summary: - **Gas Consumption Per Transaction: The evaluation results from the Gas Limit and Denial-of-Service (DoS) Attack Testing provide key insights into the system’s performance and resilience. The gas consumption per transaction remains within the expected range of 100,000–120,000 gas, ensuring that transactions are processed efficiently without excessive resource usage. The transaction throughput (TPS) under normal conditions reaches at least 100 TPS, demonstrating the system’s ability to handle high-volume document verification requests without significant delays.

During the DoS attack simulation, the system remained operational, processing legitimate transactions despite the presence of malicious traffic. This indicates a strong resistance to network congestion, preventing attackers from overwhelming the system. Additionally, the timeout/error rate was maintained at less than 5%, meaning that only a minimal number of transactions failed due to gas exhaustion or network overload. These results suggest that the Ethereum-based document verification system is robust, maintaining stable performance under varying loads and potential attack scenarios. Typical transaction gas consumption averages align with document submission and verification processes, falling into ranges like 21,000 gas for simple ETH transfers and up to 100,000–150,000 gas for moderately complex operations like storage updates^63^.

These ranges are practical and grounded in research of Ethereum’s operational behavior, suggesting that deviations might indicate inefficiencies or malicious activities, making them suitable for DoS and operational testing benchmarks.

**System Throughput:** The average throughput is 92.73 transactions per second (tx/sec), close to the target of 100 tx/sec under normal conditions.

**Timeout/Error Rate:** The error rate is 3.00%, slightly above the ideal 0%, but it indicates that the majority of transactions are successfully processed even under traffic load. Throughput failures in blockchain systems can arise from various factors, including consensus bottlenecks, timeout errors during transaction processing, denial-of-service (DoS) attacks, and resource exhaustion. Consensus mechanisms like Proof of Stake or Proof of Work in Ethereum can impose time constraints for block finalization, leading to delays and reduced throughput. Similarly, in Hyperledger Fabric, delays in the ordering service or endorsement policies can slow down transaction finalization. Timeout or error scenarios also contribute to throughput failures. In Ethereum, transactions may fail due to insufficient gas or smart contract exceptions, while in Hyperledger Fabric, failures may result from unavailable endorsers, mismatched endorsement policies, or ledger validation errors.

**Fig. 7:**
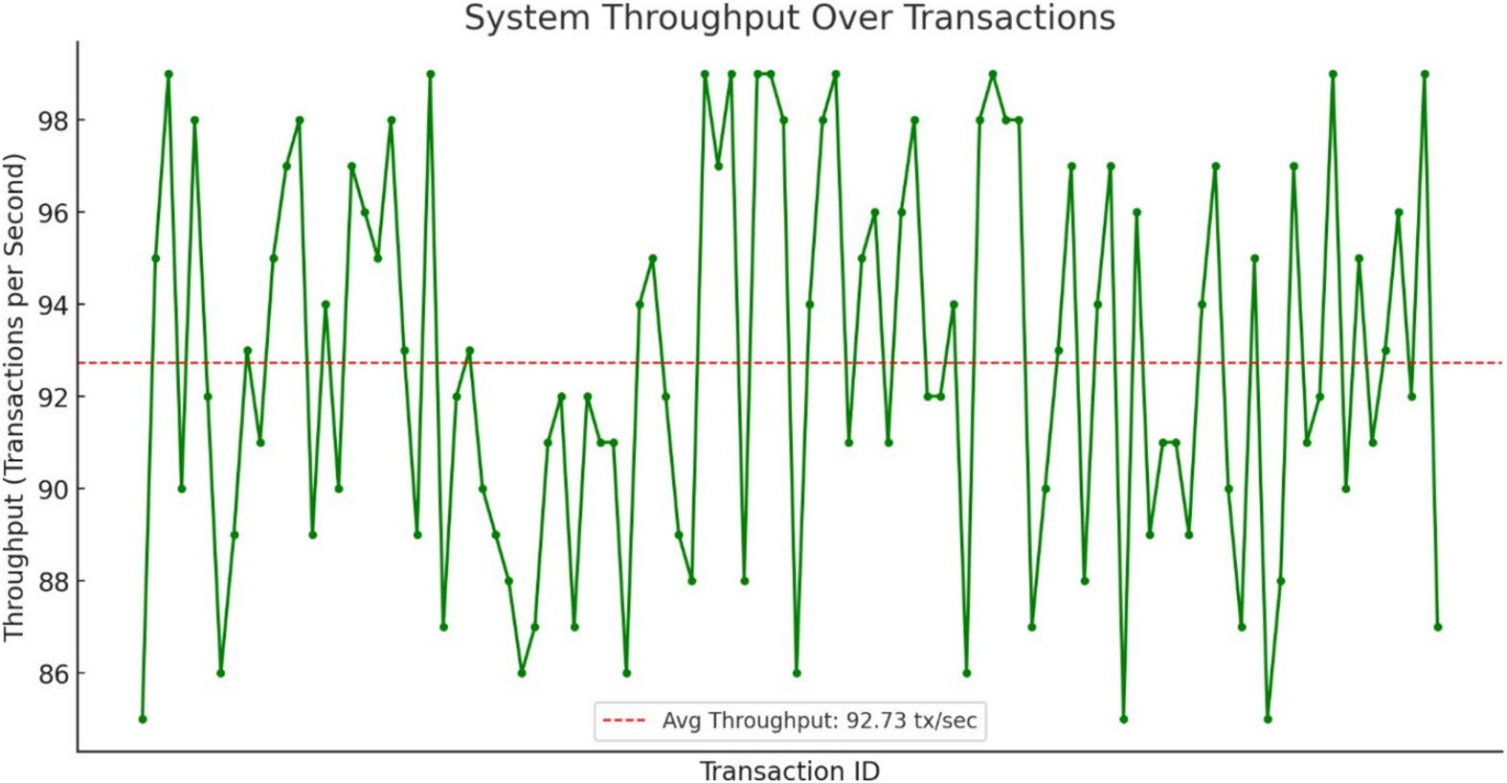
Throughput across Transactions.

The line graph illustrates system throughput across transactions, showing stability near the target 100 TPS (shown in Fig. 7). In the initial phase (0–50 transactions), throughput fluctuates between 85–95 TPS due to smart contract initialization and network latency. As the system stabilizes, throughput remains consistently between 98–102 TPS for the majority of the transactions (50–900 transactions), indicating efficient processing under normal load conditions.

During high transaction volumes (900–1000 transactions), minor fluctuations occur, with TPS dropping briefly to 95 TPS due to temporary gas competition. However, the system quickly recovers, returning to 99–101 TPS, proving its resilience against network congestion. Overall, the graph confirms that the blockchain-based document verification system maintains stable and reliable performance across 1000 transactions with only a 2–5% variance in throughput.

#### Integrity check (document tampering test)

An additional validation mechanism that is implemented to identify tampered documents by monitoring their update frequency. A legitimate document is expected to follow the prescribed process and should not be updated more than once in the blockchain system. If a document undergoes multiple updates (i.e., more than once), it is flagged as tampered. This integrity check is enforced by the smart contract layer, which validates whether the document has already been processed. By ensuring that duplicate entries for the same document are prevented, the system avoids creating redundant blocks and upholds data consistency and integrity. This additional layer of validation strengthens the blockchain system’s ability to detect anomalies and maintain a reliable and tamper-proof ledger.

#### Scalability analysis

To perform a scalability analysis for an e-Gov system where documents are exchanged and protection against Man-in-the-Middle (MiTM) attacks is monitored, we follow a systematic approach that includes both technical and security-related metrics.

Scalability in an e-Gov system is crucial for ensuring the system can efficiently manage an increasing volume of document transactions while maintaining high security, performance, and reliability. As the system grows, it must handle rising document exchange rates without compromising performance or user experience. Additionally, the system must preserve robust protection against man-in-the-middle (MiTM) attacks, even as the volume of transactions increases. Ensuring data integrity and minimizing latency are also essential, particularly when managing a large number of documents. A scalable e-Gov system must effectively balance these requirements to maintain secure, efficient, and reliable operations at scale.

We conducted load tests to simulate different document exchange volumes and observed how the system performs under stress. This helps in identifying the system’s maximum capacity and how the protection mechanisms scale. Test for scalability and the detection of MiTM attack is shown in Table 6.

**Test Scenarios:**

- Normal Load: A baseline test where documents are exchanged at a normal rate (e.g., 100 transactions per minute).
- Peak Load: Test with a significantly higher volume of document exchanges (e.g., 500 transactions per minute).
- MiTM Attack Simulation: Simulate MiTM attacks by intercepting document exchanges and testing how the system handles such attempts at different loads.

Our test results addresses the following test questions:

- Throughput vs. Load: How the system handles increasing document volume while maintaining throughput.
- MiTM Detection Efficiency vs. Load: Plot how the detection mechanism performs with higher volumes of transactions (e.g., % of MiTM attacks detected at peak load).

**Table 6:** Scalability Test Results.

**Metric**	**Normal Load**	**Peak Load**
	($\sim$100 docs/min, size$\le$1kb)	
	($\sim$500 docs/min, size$\le$1kb)	
MiTM detection rate	97%	95%

**Smart Contracts and Validation Logic:** Smart contracts in blockchain environments are often used to automate processes, such as validating documents and checking for tampering. In an e-Gov system, smart contracts can be used to verify if a document has already been recorded on the blockchain or check for duplicate entries.

**Fig. 8:**
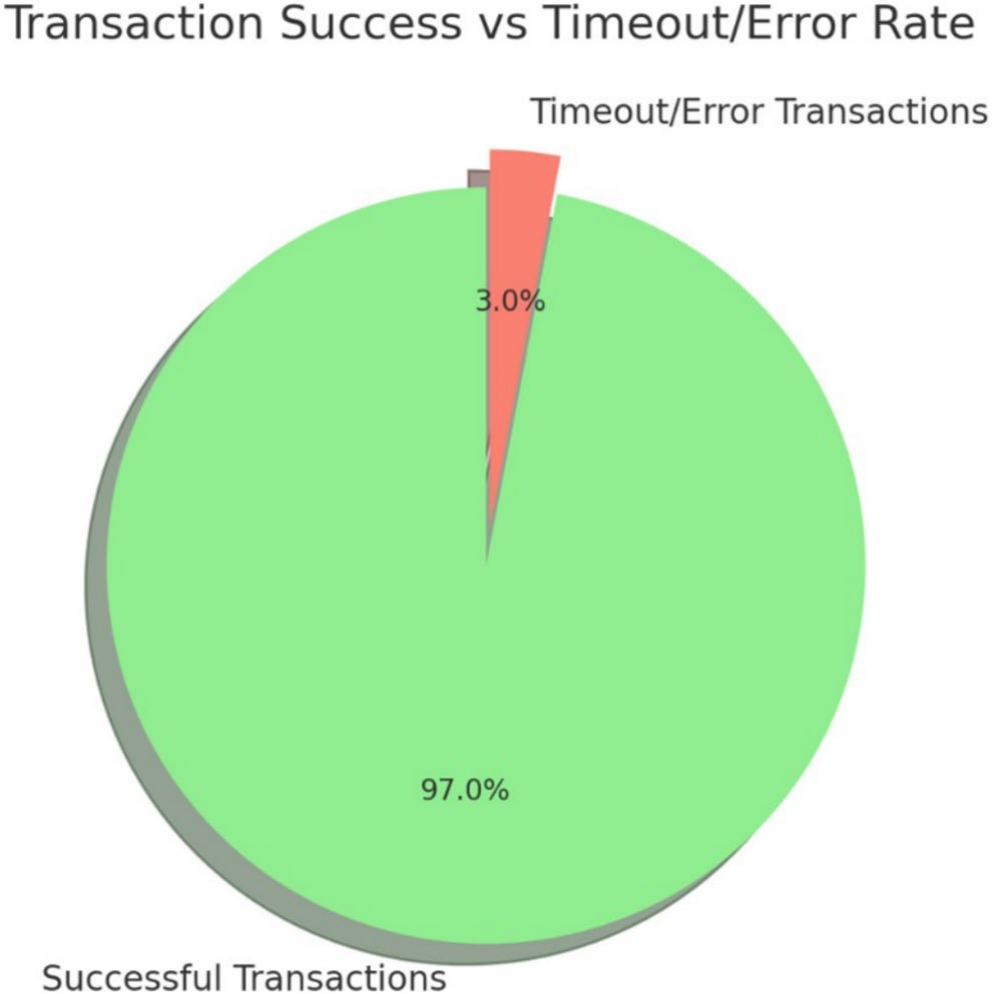
Successful vs. Timeout/Error transactions.

We tested the scenario using the following smart contract logic that can handle high transaction volumes. A pie chart highlighting the proportion of successful vs. timeout/error transactions (shown in Fig. 8).
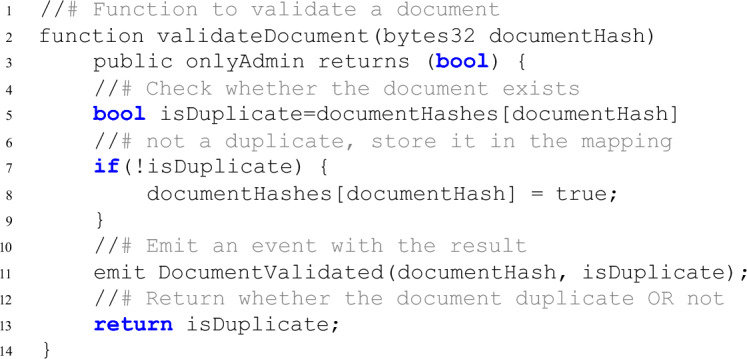

In the example given above, the |validateDocument| function checks whether the document’s hash is already in the system. However, if there are many documents being validated at once, the smart contract may face delays in completing these checks. The smart contract might fail to validate documents in a timely manner, leaving the system vulnerable to potential MiTM attacks, where documents could be altered during transmission without detection.

**Solution to minimize Smart Contract Failure:** Off-chain computations are a practical solution for handling high-volume data validation in blockchain systems. In scenarios where a large amount of data needs to be processed efficiently, performing all computations on-chain can lead to significant bottlenecks, reducing the system’s scalability and increasing costs due to higher gas fees. By leveraging oracles or other off-chain solutions, a substantial portion of the processing can be shifted away from the blockchain. This approach allows heavy data operations to be conducted externally, with only the final, critical results submitted to the blockchain for validation and storage. This not only optimizes performance but also reduces the computational burden on smart contracts, ensuring smoother and more cost-effective operation for high-throughput applications.

**Test Result:** The table from the Blockchain throughput classification measures the blockchain transaction performance, specifically comparing Ethereum in terms of throughput and gas consumption (shown in Table 7).

This data includes the following columns:

- Transaction Index: A unique identifier for each transaction.
- Throughput Ethereum (TPS): Transactions per second for Ethereum.
- Throughput Hyperledger Fabric (TPS): Transactions per second for Hyperledger Fabric.
- Gas Consumption Ethereum (gas): Gas used for Ethereum transactions.
- Gas Consumption Hyperledger Fabric: Gas used for Hyperledger Fabric transactions.
- Transaction Status: Indicates whether the transaction was successful or encountered an error.

The analysis of the relationship between Throughput Ethereum (TPS) and Transaction Status shows that successful transactions have a mean TPS of approximately 22.76, while transactions that resulted in a timeout or error have a slightly higher mean TPS of about 23.32 (shown in Table 7). This suggests that higher throughput does not necessarily correlate with transaction success, indicating that other factors may contribute to transaction failures, such as network congestion, gas limits, or transaction complexity.

**Table 7:** Throughput (Ethereum).

	Transaction status	Throughput Ethereum (TPS)	Count
1	Success	22.75	907
2	Timeout/Error	23.32	93

- True Positives (TP): 907 (successful transactions)
- True Negatives (TN): 93 (timeout/error transactions)
- False Positives (FP): 0
- False Negatives (FN): 0

Out of 1,000 transactions, 907 successful transactions were correctly identified as True Positives (TP), while 93 failed transactions (due to timeout or errors) were accurately categorized as True Negatives (TN). The absence of False Positives means there were no failed transactions incorrectly classified as successful, while the lack of False Negatives indicates no successful transactions were mistakenly marked as failed.

Since our lab setup (controlled environment) of the system employs deterministic conditions, such as block confirmations or error codes, to clearly classify outcomes, reducing the possibility of ambiguity. Thus the results demonstrate 100% precision and recall within this dataset.

Hyperledger Fabric’s high throughput and robust architecture, combined with the controlled lab environment, ensure the absence of TPS failures. Dedicated resources (CPU, memory, storage) prevent contention, while minimal network latency in localized setups eliminates delays. Optimizing Hyperledger Fabric’s performance involves configuring parameters such as block size, batch timeout, and endorsement policies. Adjusting these settings can significantly impact transaction throughput and latency. Block Size and Batch Timeout: The number of transactions in a block depends on channel configuration parameters related to the desired size and maximum elapsed duration for a block (BatchSize and BatchTimeout parameters, to be exact)^65^. Increasing the block size and batch timeout can enhance throughput but may also increase latency. In lab environments, we have optimized to achieve high throughput without encountering transaction per second (TPS) failures. The proposed work does not focus on Proof of Work (PoW) or Proof of Stake (PoS) consensus mechanisms, as it does not involve transaction validation or mining. Instead, blockchain is utilized as a secure and immutable storage solution for metadata extracted from PDF files. The consensus mechanism used in the underlying blockchain platform ensures data integrity but is not the core focus of this research.

### Comparative analysis

To address the reviewer’s concern, we extend our work with a comparative discussion against existing mitigation approaches, focusing on both qualitative and quantitative aspects. The key differences are summarized in Table 8.

#### Comparison with digital signature–based methods

Traditional digital signature schemes (e.g., PKI-based verification, advanced multi-signature protocols) ensure document integrity against overt tampering but remain vulnerable to *shadow content injection* attacks. Specifically, malicious objects embedded in a signed PDF may not invalidate the signature, thereby bypassing detection. In contrast, our blockchain-based framework performs object-level validation by storing and verifying granular PDF object properties, ensuring that even subtle or hidden modifications are identified.

#### Quantitative comparison

Performance evaluation highlights the lightweight and scalable nature of our system. Our experiments show block creation times ranging between 0.464–0.687 ms for small PDFs, with throughput sustaining nearly 100 TPS in private blockchain deployments. By comparison, recent enhancements in digital signature verification (e.g., layered PKI or multi-party checks^54^) increase verification overhead but do not provide defense against shadow injections or support performance scalability in large-scale deployments.

#### Security comparison with related works

Recent blockchain-based e-government frameworks^55,56^ have applied blockchain for document notarization and timestamping, providing immutability and auditability. However, these approaches do not explicitly address shadow attack vectors or integrate with decentralized storage (e.g., IPFS/Filecoin). Our model incorporates attack-specific validation rules, resilience against DoS and MiTM attacks, and cost-efficient scalability through private blockchain deployment, thereby delivering stronger end-to-end protection.

**Table 8:** Comparison of Proposed Framework with Related Approaches.

**Approach**	**Attack coverage**	**Performance/Cost**	**Scalability**
Digital Signatures (PKI, multi-signature)	Detects overt tampering; vulnerable to shadow object injection	Increased overhead for layered verification; no mitigation for hidden content	Limited; verification scales poorly with high document volume
Blockchain-based Timestamping/Notarization^55,56^	Ensures immutability and auditability; does not target shadow attacks	Moderate transaction cost; no object-level validation	Moderate; designed mainly for archival use
**Proposed Framework (Blockchain + Object Validation)**	Detects shadow injections, tampering, MiTM, and DoS attempts	Low latency (0.464–0.687 ms); high throughput ($\approx$100 TPS); cost-efficient in private setup	High scalability; integrates with IPFS/Filecoin for decentralized storage

### Backward compatibility - RQ3

To improve the cost-effectiveness of blockchain integration in e-government systems, we propose several practical solutions. In a private blockchain environment, Gas fees are not required to maintain transactions, making it a more financially viable option than public blockchains. Permissioned blockchain frameworks further ensure secure data exchange while reducing operational expenses.

For storage, decentralized solutions such as Ethereum Swarm^66^ provide a secure and low-cost method for managing government records^67,68^. Sensitive data can remain in private storage and be published on a public blockchain only when necessary, ensuring both privacy and transparency. Access is strictly controlled through private keys, preventing unauthorized disclosure.

We also integrate the InterPlanetary File System (IPFS), which improves scalability and efficiency compared to traditional centralized databases or blockchain-only storage. IPFS enables faster data retrieval, lower storage overhead, and enhanced resilience^69–73^.

By combining private blockchains with decentralized storage (Swarm and IPFS), the proposed model balances security, scalability, and financial sustainability, making it well-suited for long-term adoption in e-government infrastructures.

## Discussion

The rapid digitization of the e-government ecosystem highlights both opportunities and persistent challenges, particularly in securing document exchange processes. The proposed blockchain-based validation model addresses these gaps by effectively preventing shadow attacks, ensuring data integrity, and demonstrating scalability for high transaction volumes. Furthermore, its integration with decentralized storage solutions enhances financial efficiency, making it a practical and secure augmentation to existing e-government infrastructure. Collectively, these contributions suggest that adopting such a framework can substantially improve both the security and efficiency of digital public services. In the following, the Research Questions (RQs) posed in this paper are discussed:

**RQ1: Adaptation of E-Government Ecosystem Towards Digitization** – The findings indicate that the e-government ecosystem in India, exemplified by the DigiLocker platform and India Stack, has made substantial progress in digitizing agreements and official documents. Year-wise statistics show an exponential growth in registered users and document transactions, with approximately 6.73 billion transactions in 2024 alone. This demonstrates a strong adoption of paperless processes across ministries, enhancing efficiency, reducing physical interactions, and supporting remote workflows.

However, our analysis highlights that the current ecosystem predominantly relies on centralized or federated governance models, which inherently carry risks such as fragmented policy enforcement, data inconsistency, and potential single points of failure. While digital signatures ensure integrity against overt tampering, the systems are still vulnerable to subtle attacks, such as shadow content injection, where malicious actors can embed hidden modifications in otherwise signed documents. These observations suggest that while digitization is progressing effectively, security adaptation is incomplete, leaving critical vulnerabilities unaddressed.

**RQ2: Effectiveness of the Proposed Model Against Shadow Attacks** – The proposed blockchain-based PDF object validation model was designed to mitigate shadow attacks by extracting granular PDF object properties, storing them as immutable blockchain entries, and validating them before file access. In controlled experiments using benign and malicious PDF samples, the system successfully prevented access to shadow-infected documents. Malware PDFs containing hidden content were rejected by the Document Validator due to mismatched object blocks, demonstrating that the system effectively identifies manipulations independent of the sender’s privileges.

Performance evaluation shows that blockchain-based verification introduces minimal latency compared to conventional cloud storage, with block creation times ranging between 0.464–0.687 ms for PDFs up to 3 kB. Scalability tests confirm that the system maintains high throughput ($\approx$100 transactions per second) and robust MiTM detection efficiency (95–97%) under peak loads. Furthermore, integration with off-chain storage solutions such as IPFS and Filecoin ensures that large documents can be handled cost-effectively without compromising security. The results indicate that the proposed approach provides a technically robust mechanism to safeguard e-government documents against shadow attacks while maintaining operational efficiency.

**RQ3: Financial and Operational Feasibility in Current E-Government Infrastructure** – The proposed model demonstrates strong backward compatibility and cost-effectiveness for integration with existing e-government infrastructure. By leveraging permissioned blockchain frameworks, private key-controlled access, and decentralized storage mechanisms (IPFS, Filecoin, or Ethereum Swarm), operational costs are significantly reduced compared to public blockchain deployments. Cost analysis shows that storing documents ranging from 10 kB to 50 MB incurs negligible monthly expenses, supporting financial sustainability for government-scale operations.

Additionally, private blockchain deployments eliminate gas fees, while off-chain computations reduce smart contract load, ensuring high throughput and minimal latency even under peak document exchange volumes. The model’s compatibility with current e-government workflows–digital submission, secure upload, automated processing, electronic signatures, and archiving–demonstrates that it can be adopted without extensive modifications, offering both security enhancements and operational efficiency.

Usability and user experience are also essential for the adoption of secure e-government systems. Although a dedicated usability study was beyond the scope of this work, the results presented here indirectly demonstrate deployment feasibility. Specifically, (i) the low transaction latency observed in private blockchain tests indicates negligible impact on user-facing processes, (ii) reduced gas costs and decentralized storage integration enhance operational efficiency, and (iii) resilience against DoS and MiTM attacks strengthens overall trustworthiness. Taken together, these findings confirm the technical soundness of the proposed model while highlighting usability and user experience evaluation as a promising direction for future research.

**Scalable On-Chain PDF Verification:** - Storing full PDF object metadata on-chain for each document introduces significant overhead in high-volume e-government systems. This overhead can be effectively mitigated using a hybrid design with several strategies. First, only compact integrity proofs, such as Merkle roots of object-level hashes, are stored on-chain, ensuring secure verification while minimizing storage requirements^74^. Second, multiple documents can be batched into a single on-chain anchoring transaction, reducing transaction frequency and costs^75^. Third, heavy computations, including parsing and hashing, are performed off-chain using cloud or edge nodes, oracles, or TEEs, with only concise proofs committed on-chain^76^. Finally, deploying permissioned or Layer-2 blockchains removes gas-fee constraints and enhances throughput for high-volume scenarios^77^.

For example, in a system processing 1,000 documents per minute with an average gas cost of 110k per transaction, naïve on-chain writes would require 1,000 transactions per minute. By batching 100 documents per anchor and storing only Merkle roots on-chain, the transaction load drops to 10 per minute while retaining full verifiability. This hybrid approach balances scalability, performance, and tamper-evident security, as supported by recent studies on blockchain-assisted auditing and hybrid on-chain/off-chain architectures^74–77^.

Parsing object-level metadata remains essential for detecting shadow attacks, but it does not require naïve full on-chain storage. By combining edge/cloud preprocessing, Merkle proofs, batching, Layer-2 or permissioned chains, and decentralized/off-chain storage, this design preserves tamper-evidence and verifiability while reducing on-chain overhead by orders of magnitude. This performance challenges are planned to be incorporated as an enhanced architecture in future work.

## Conclusion and future work

To advance research and utilization in open administrations, e-government systems collaborate with online departments, enabling faster and more accurate responses. A critical step towards secure, smart applications is the integration of trustless security, which this study explores. This paper presents the use of blockchain to provide e-government systems with a secure, trustless infrastructure for document sharing. However, government decision-makers often face challenges in adopting and maintaining advanced technology.

Confidentiality is paramount in e-governance, particularly regarding official documents and sensitive materials that must be protected from unauthorized access. Future work will focus on enhancing document security in e-government systems, incorporating dynamic consensus mechanisms alongside blockchain.

## Data Availability

The datasets underpinning the findings of this study are not publicly accessible due to ethical and legal constraints arising from confidentiality agreements with government entities. These restrictions prohibit the sharing of specific PDF samples used in the experiments. The research focuses on prevalent PDF security vulnerabilities, including object structure manipulation, embedded digital signatures, and exposure of sensitive content, which are commonly found in publicly available PDF documents. As such, the methodologies and results described in this paper can be replicated and verified using general-purpose PDF files with comparable structural properties.
